# A review of recent advancements in the impact response of fiber metal laminates

**DOI:** 10.1016/j.heliyon.2025.e41756

**Published:** 2025-01-07

**Authors:** Vijayan Muniyan, Vishnu Vijay Kumar, Indran Suyambulingam, Suganya Priyadharshini, Divya Divakaran, Sanjay Mavinkere Rangappa, Suchart Siengchin

**Affiliations:** a Department of Mechanical Engineering, Coimbatore Institute of Technology, Coimbatore, 641014, India; b Department of Aeronautical Engineering, School of Mechanical Engineering, Sathyabama Institute of Science and Technology, Chennai, 600119, India; c Structural Engineering, Division of Engineering, New York University Abu Dhabi (NYUAD), PO Box 129188, Abu Dhabi, United Arab Emirates; d Sophisticated Testing and Instrumentation Centre (STIC), Department of Mechanical Engineering, Alliance School of Applied Engineering, Alliance University, Bengaluru, Karnataka, 562106, India; e Department of Biotechnology, Saveetha School of Engineering, SIMATS, Chennai, 602105, Tamil Nadu, India; f Natural Composites Research Group Lab, Department of Materials and Production Engineering, The Sirindhorn International Thai-German School of Engineering (TGGS), King Mongkut's University of Technology North Bangkok (KMUTNB), Bangkok, 10800, Thailand

**Keywords:** Fiber metal laminates (FMLs), Impact response, Low-velocity impact (LVI), High-velocity impact (HVI), Composites

## Abstract

Fiber metal laminates (FMLs) have garnered significant attention due to their exceptional impact resistance, making them attractive for various structural applications. This review presents recent advancements in understanding the impact behavior of FMLs under low- and high-velocity impact scenarios. Low-velocity impacts, commonly encountered during manufacturing, handling, and tool drops, are discussed, with a focus on damage mechanisms, energy absorption capabilities, and influential factors such as impactor geometry and boundary conditions. Additionally, this review delves into high-velocity impact events, simulating scenarios such as ballistic impacts, highlighting the role of FMLs in mitigating perforation and enhancing damage tolerance. The effects of various parameters on the impact response are critically analyzed. The findings presented herein contribute to the development of lightweight, impact-resistant FML components for aerospace, automotive, and defence applications.

## 1 Introduction

Significant milestones in the evolution of an aircraft's principal structure include the use of composites (fiber polymers) in the 1970s and 1980s, as well as the transition from wood to aluminum in the 1930s. Another notable advance occurred in the 1950s and 1960s with the introduction of fiber-reinforced polymers (FRPs), which include carbon, aramid, glass, and boron fibers known for their strength and stiffness. However, since fibers cannot act as autonomous structural components, they are often wrapped in a plastic matrix, yielding fiber-reinforced polymers (FRPs) [1]. Fiber alignment must match the load direction while producing FRP materials. Fiber metal laminates (FMLs) consist of a continuous matrix, distributed reinforcements such as fibers and particles, and an interlayer area. These FRP materials are joined with thin metal sheets to form FML composites. FMLs are typically manufactured using the hot-pressing technique, which also cures the layered composite and shapes the metal layers into the necessary forms. Several variants are currently available, including GLARE (glass fibers and aluminum), CARALL (carbon fibers and aluminum), ARALL (aramid fibers and aluminum), and TIGR (titanium and graphite fibers).

As a type of hybrid composite, fiber metal laminates combine the best features of both metal and composite materials. The structure consists of thin aluminum sheets that possess great strength and are affixed in an alternating manner to layers of FRP epoxy adhesive [2,3]. ARALL (aramid fiber reinforced aluminum laminate) is the term given to the first generation of laminates made from aramid fibers, and GLARE (glass fiber laminate aluminum reinforced laminate) is given to the second generation of laminates made from high-strength glass fibers [4]. GLARE was created in a unidirectional variant as the strength of fibers aligned in one direction and biaxial forms as GLARE 3 in the range of 0–90 directions [5]. Since the metal and the material combination are mixed together, these hybrid stacks are very stable at high temperatures and can be used under high-temperature conditions [6]. The classifications of FML as ARALL, GLARE, and CARALL were discussed by Sinmazçelik et al. [7], who also discussed the significance of its main components, metals, and fiber-reinforced laminates. One notable feature of these materials is their superior fatigue resistance in comparison to that of conventional laminates that use mechanical bonding. Katnam et al. [8] reported that bonded laminates exhibited enhanced stress properties. These laminates are known for their ability to efficiently distribute stress during fastening and riveting, excellent fatigue resistance, and compatibility with a wide variety of materials. According to da Silva & Campilho [9], the use of fiber-bonded laminates reduced the need for mechanical pins in laminate structures.

**Fig. 1:**
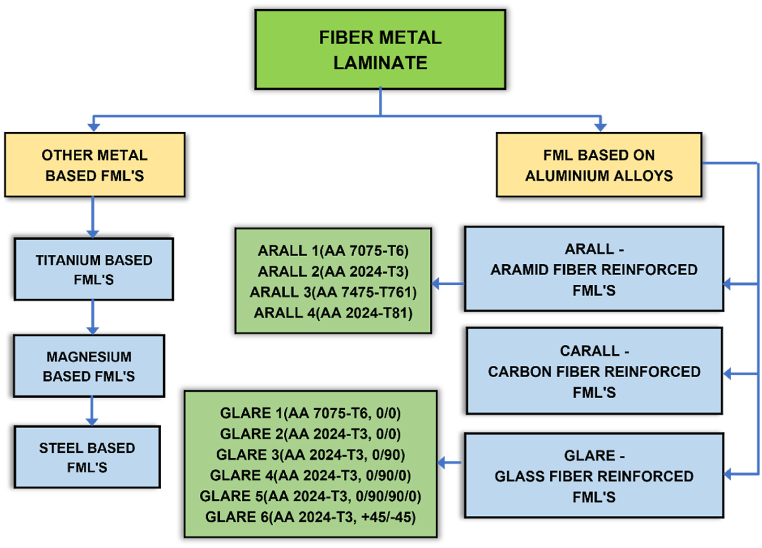
FML classification.

FMLs are categorized according to the metals and fibers employed in their fabrication. Based on the type of fiber used for laminates with aluminum fabrication, FMLs are classified as ARALL, CARALL, or GLARE, as shown in Fig. 1. Glass fiber is utilized to create GLARE laminates, carbon fiber is used to make CARALL laminates, and Aramid fiber serves as the primary reinforcement for ARALL. GLARE is further classified as GLARE 1–6 according to the direction of the fibers within the laminate. Similarly, ARALL is also classified from ARALL 1 to ARALL 4. Other FMLs that alternate with aluminum are titanium-based FMLs and magnesium-based FMLs. FML is appropriate for lower wing skin panels on Fokker 27, A380 fuselages as bottom wing skins, leading edges and stabilizers on wings, and Boeing cargo doors. The Airbus A380 is GLARE's most notable success in airframe construction. GLARE 2 was used for the fuselage strapes. GLARE 5 laminates are utilized in the cargo bay floors, radar reflector front bulkhead, and cargo planes. Glare 2 was used in the engine cover of Lockheed by glued patch repairs. All of the aerofoil surfaces in the A380 tail are made of GLARE, which has greater impact resistance than aluminum. It has been recommended that passenger flooring, flat or curved fuselage bulkheads, wing skin, fuselage frames, firewalls, and cargo barriers be employed.

**Fig. 2:**
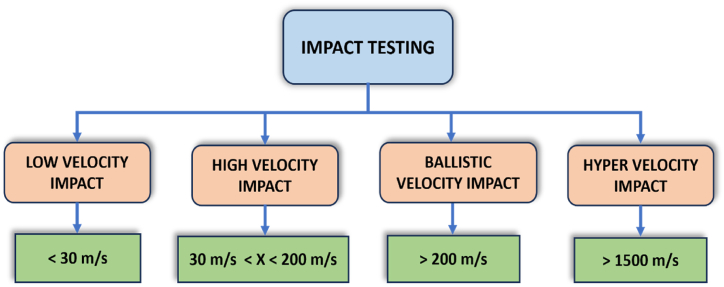
Classification of impact loading based on impact velocity.

The impact on aircraft components is a crucial factor that significantly influences the strength and durability of the materials utilized. Factors such as runway debris, tools, birds, hailstorms, and wind gusts can cause damage to aircraft parts. The impacts are divided into three categories: low-velocity, high-velocity, and hypervelocity impacts. A drop mass impact testing machine with a heavy mass impact will perform low-velocity impact. The drop mass provides the lowest range of 0–30 m/s below the velocity of freely falling objects. High-velocity and ballistic impact loads ranging from 30 to 200 m/s are obtained with pneumatic guns or other lab equipment. Ballistic velocity impact loading ranges from 200 to 1000 m/s. Hypervelocity impact loading is utilized in traditional firearms, wherein the combustion of the propellant occurs posterior to the projectile, leading to the generation of muzzle velocities greater to 1500 m/s, The categorization of impact loading is visually represented in Fig. 2.

For structures requiring a high level of safety, such as those used in aircraft, understanding a structure's dynamic response and damage resistance is crucial. A significant number of impact tests have been conducted on flat plates with either simply supported or clamped borders [10]. Material-based characteristics and geometry-based factors are the two main types of variables that affect how FML responds to low-velocity strikes. The stacking arrangement, fiber volume ratio, and interface bonding are all material-based properties. Examples of materials include metals and fibers. The parameters that are based on geometry include specimen stretching, the effect of specimen size and impactor mass, and velocity.

## 2 Low-velocity impact testing on FML

Various approaches, such as dropping weight, Izod, Charpy, and ballistic impact, can be used to perform low-velocity impact tests. Both the Izod and Charpy impact test procedures generate impact from a mass that is swinging. The kinetic energy and velocity of the impacting mass are modified, which transmits energy and causes work to be done on the specimen. The energy absorption by the specimen occurs through kinetic energy acquisition, deformation, resistance between the specimen and impactor, and hysteresis effects. Both tests are often used to compare how isotropic materials with various compositions respond to impacts. To concentrate stress and reduce the energy needed to initiate a fracture, tests are performed on the specimens. In the LVI impact study, the failure mechanisms identified for FMLs include delamination occurring at both the metal-fiber interface layer and within the composite layers. These delamination phenomena are mostly attributed to plastic deformation in the metal and the presence of high stress levels [11]. Impact-related damage mechanisms include fiber cracks and metal layer cracking, which initiate laminate perforation depending on the impact energy [12]. The failure of a specimen due to LVI involves the absorption of strain energy, the formation of cracks in the matrix, and the occurrence of fiber fracture and delamination.

### 2.1 Aluminum-based FMLs

**Fig. 3:**
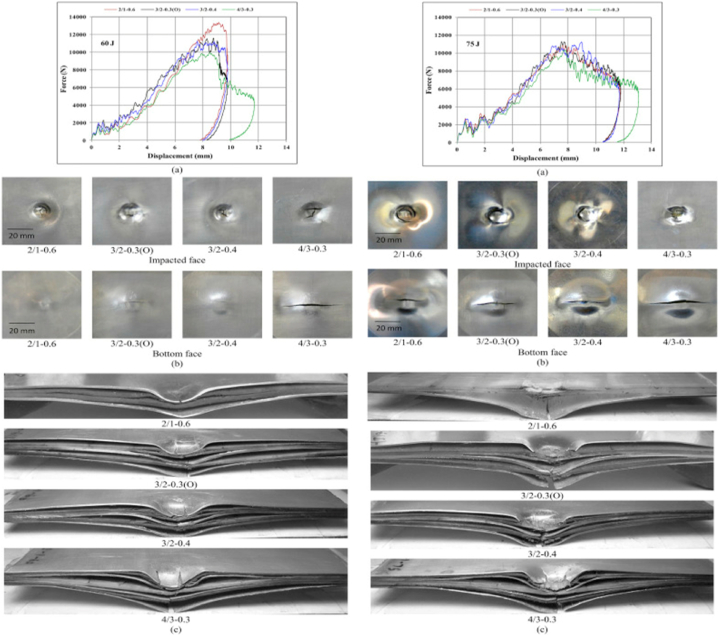
a)Force Displacement Curves FML at 60 J, and 75 J, b) Failures on top and bottom surface layers, c) Sectional View of failed FML [13]. (Reproduced with permission from Elsevier License Number – 5604030015245).

Low-velocity impact tests were conducted by varying the properties of the aluminum layer distributions. Sharma et al. [13] investigated the influence of the aluminum layer distribution on the LVI impact response on FMLs. Using drop-weight testing equipment, they applied low-velocity impact loads to FMLs. The fabrication of the FMLs involved the use of AA 2024 and GFRP, employing vacuum bagging techniques. The spatial arrangement of the metallic layers varied with respect to the thickness of the four alternating layers studied but maintained a consistent thickness. The efficiency of the FMLs was compared using the first cracking energy, maximum displacement, maximum force, fracture length, degree of damage, and permanent deformation. Force displacement curves, surface layer failures, and sectional views of the FML at 60 J, and 75 J are shown in Fig. 3a – c.

Using aluminum alloy 2024 sheets and S-glass fiber/epoxy composite layers, Caprino et al. [14] conducted LVI experiments on aluminum/glass-fiber composites using a drop-weight impact machine. To facilitate comparison, identical tests were conducted on monolithic aluminum sheets with matching thicknesses. The authors found that the energy needed to cause initial failure is extremely low, whereas the impact energy that causes fiber failure is comparable to the impact energy level that causes initial cracking in aluminum sheets.

Compared to carbon fiber- and GFRP-based materials, fiberglass-aluminum appears to respond to complete penetration more efficiently. However, a monolithic aluminum sheet of similar thickness prevents penetration better than a fiberglass-aluminum laminate. Lin et al. [15] used numerical and experimental approaches to examine the LVI resistance of FML (AA/GF/PP) laminates with varying bonding properties between metals and composites. Aluminum alloy sheets that had undergone surface treatment, such as nitrogen plasma and phosphoric acid anodizing, were used to create laminates of aluminum sheets, glass fibers, and polypropylene fibers. The results demonstrated that under impact energy levels of 20 J and 30 J, delamination was the primary route of failure for the two laminates tested. The increase rates and percentages of residual displacement in laminates that underwent modified plasma surface treatment under increasing impact energy were slower than those in laminates that underwent anodized surface treatment.

In the research by Sisan & Eslami-Farsani [16], multiple lay-up arrangements of FMLs were constructed and analyzed under LVI loading conditions. Glass, Kevlar, and carbon fibers were mixed with 2024-T3 aluminum sheets in various combinations. Using an optical microscope, the damaged specimens were studied, and the force‒time profiles of the impact forces were noted.

Laliberté et al. [17] conducted experimental research to investigate impact resistance, specifically examining the effects of LVI tests on commonly accessible GLARE laminates. Glare laminates 3-2/1, 4-2/1, and 5-2/1 underwent a thorough series of impact tests. Using the developed impact test method, the relative impact performance of commercially available Glare laminates was evaluated. Due to Glare5 having a greater fiber volume fraction than AA 2024-T3, Glare3, or Glare4, Glare5 exhibits greater energy absorption and sustains less damage per unit area when subjected to impact. Consequently, it appears to be better suited for scenarios where impact damage holds significant design significance.

**Fig. 4:**
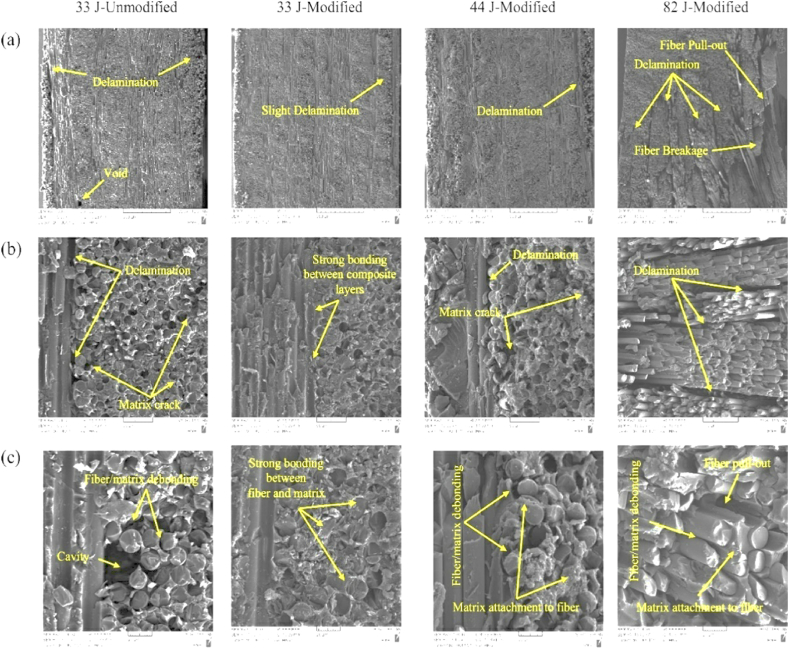
Comparison of modified and unmodified SEM images of failed FMLs: (a) cross-sectional area and (b) and (c) magnified images of the damaged section [18]. (Reproduced with permission from Elsevier License Number – 5604021275085).

Fathi et al. [18] conducted trials to assess the effect of including GNPs on the LVI behavior of FML composites. For FML panels with a 2/1 design, the impact response and damage pattern of untreated and modified specimens exposed to various impact energies are evaluated. The results reveal that adding 0.2 wt% GNPs to FMLs enhances the impact resistance, with treated panels having greater bending stiffness, peak load, energy absorption, and SEA than unreinforced panels. The findings suggest that the incorporation of a weight percentage of approximately 2 % graphene nanoplatelets (GNPs) into fiber metal laminates (FMLs) leads to an increase in their impact resistance. Fig. 4a-c displays detailed FESEM images of both the modified and unmodified FMLs after impact.

The FML panels with aluminum grade 7475 and UD glass fibers/epoxy aligned in a 0°/90° configuration that were exposed to LVI were examined by Tsartsaris et al. [19]. This allowed for the development of the panels' best designs for impact-resistant aircraft applications. The experiments showed that localized plastic deformation and layer-interface failure allow FML laminates to absorb energy. Specifically, delamination was observed between successive layers of epoxy reinforced with fibers, as well as on the opposite side of the aluminum sheet and its adjacent layer. Bagnoli et al. [20] conducted a thorough assessment of the performance of GLARE laminates, which were affixed to a high-strength aluminum alloy via adhesive bonding. This evaluation covered multiple aspects, including the initiation, progression, and types of failure related to both impact and fatigue loading. The researchers used an ultrasonic C-scan approach in conjunction with metallography and SEM to acquire a detailed understanding of the material's behavior.

**Fig. 5:**
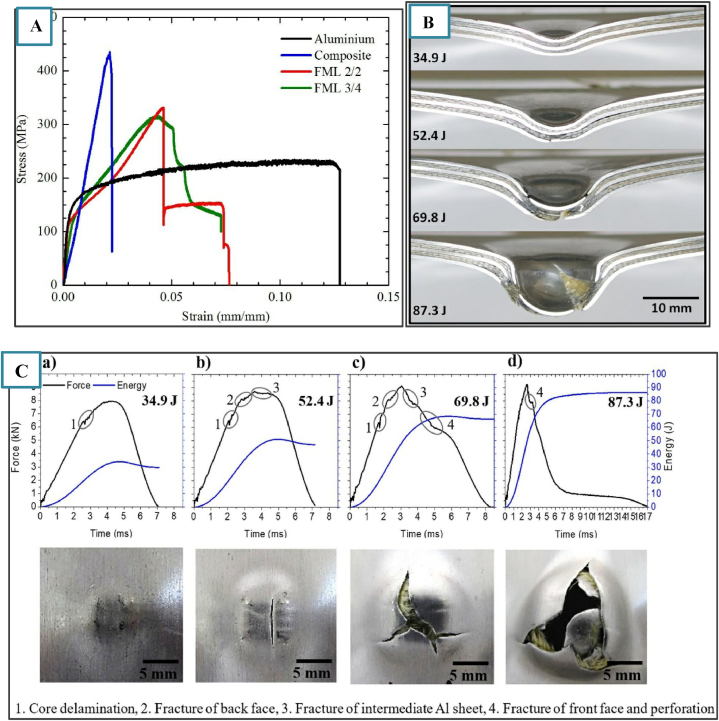
A) Stress‒strain plot from the tensile test, B) cross-sectional view of the failed FML, and C) force‒time and energy–time curves for FML 2/2 at various impact energies(a-d) and the corresponding back side of the specimen [21]. (Reproduced with permission from Elsevier License Number – 5581750432209).

Some of the authors carried out repeated LVI on FML. Yarmohammad Tooski et al. [22] examined the significance of the impact distance on the laminate response, damage, and energy absorption of GLARE 5 laminates. We can observe several parameters, such as plastic deformation of the metal, strain hardening of the composite, and perforation of the GLARE laminate, when the impacts are repeated. The importance of the metal component becomes clear when considering the spacing between successive impact locations. Carrillo et al. [21] evaluated the response of two thermoplastic FMLs to low-velocity impacts, one based on aramid fiber-reinforced polypropylene and the other based on AA 5052. The results obtained from the impact tests suggest that FMLs possess a superior perforation threshold compared to both plain aluminum and composite materials. This indicates that FMLs are more resistant to penetration, requiring more impact energy to be breached. When assessing impact performance by normalizing the absorbed energy relative to the areal density, the composite material surpasses the FML 2/2 configuration. However, among all the materials studied, the FML 3/4 arrangement has a significant impact on the performance. The stress‒strain graph for FML is shown in Fig. 5A and cross-sectional images of failed FML are shown in Fig. 5B. The force‒time plot and the rear side of the laminate are depicted in Fig. 5C(a-d).

**Fig. 6:**
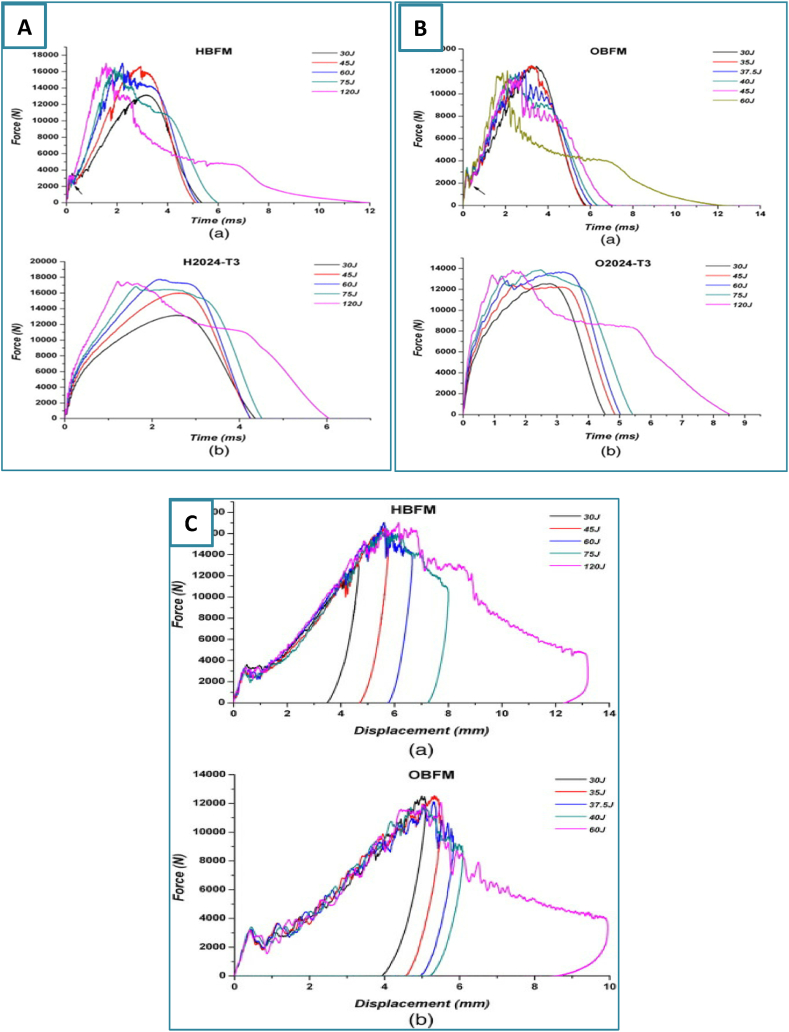
A) Force‒time for the a)HBFM, b) H2024-T3, B) Force‒time for the a)OBFM, b)O2024-T3, C) force‒displacement curves for the a)HBFM and b)OBFM [23]. (Reproduced with permission from Elsevier License Number – 5604000802610).

Ferrante et al. [23] focused on the response of FML composites composed of stacked layers of AA sheets and basalt fiber prepreg to LVI testing. This research compares these findings with those from other GLARE materials. The impact energy levels were deliberately chosen to induce both an initial crack and the thickness of the crack in the materials, but the size of the impactor was also taken into account. The findings indicated that the behavior of basalt-based fiber metal laminates (FMLs) was notably impacted by the dimensions of the impactor. Furthermore, the reaction of these laminates to impact was quite positive, demonstrating competitive performance compared to monolithic aluminum plates. The Force‒time for the HBFM, H2024-T3 FML are shown in Fig. 6A (a, b). The force‒time for the OBFM, O2024-T3 FML are shown in Fig. 6B (a, b), and force‒displacement curves for HBFM, OBFM FML are shown in Fig. 6C (a, b). Nassir et al. [24] conducted impact tests on FMLs composed of an aluminum alloy (AA2024-T3) and an S-glass fiber-reinforced polyether ketone-ketone (GF/PEKK) composite under low-velocity impact. This study aimed to analyze the effect of different stacking orders on the rate of strain on the penetration resistance of these FMLs. The study's results underscore the notable ductility exhibited by the Fiber Metal Laminates that are constructed using the Polyether ketone-ketone (PEKK) resin.

**Fig. 7:**
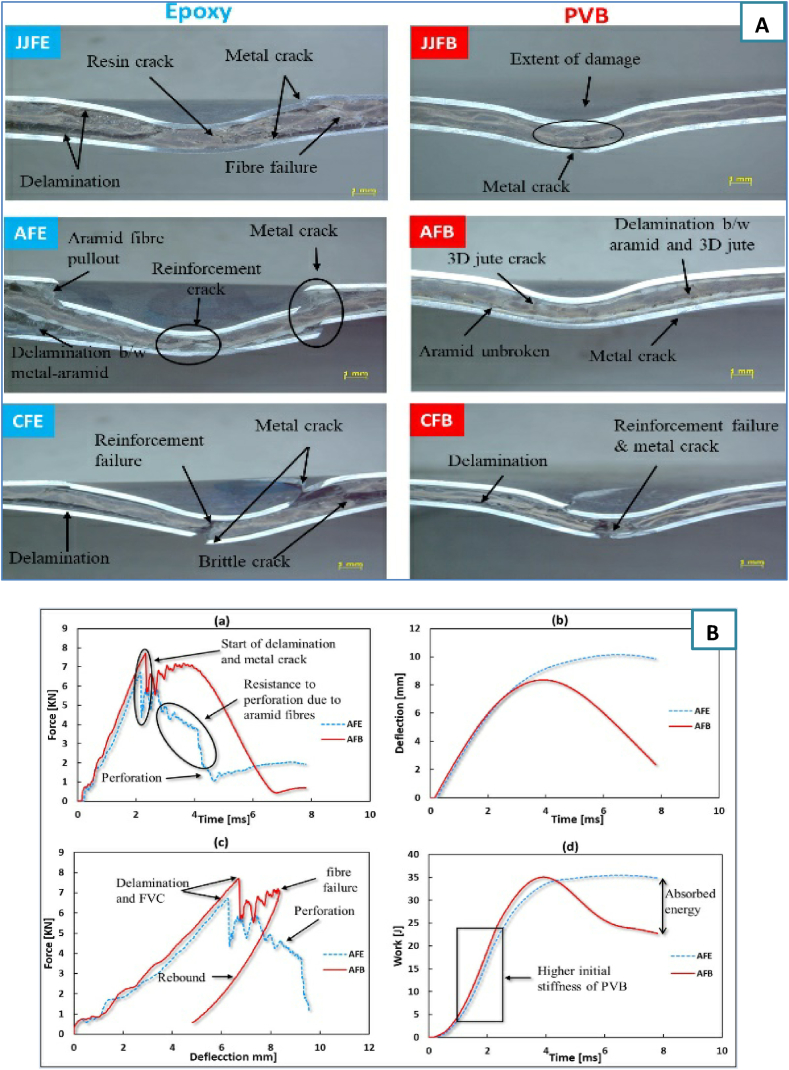
A) Cross-sectional images of failed samples of different FMLs. B) comparison of the AFE and AFB hybrid FMLs a) Force‒time, b) deflection‒time, c) force displacement, and d) work-time curves [25]. (Reproduced with permission from Elsevier License Number – 5604010270016).

Hussain et al. [25] focused on the impact of the matrix material on the LVI behavior of FML. PVB and epoxy are the two distinct materials that make up the matrix utilized in this investigation. Two plain-woven skins are layered on top of a 3D-woven jute core to form the reinforcing structure. This research aims to analyze how the choice of matrix material affects the LVI response of FMLs, particularly in conjunction with the specific reinforcement configuration involving a 3D-woven jute core. Jute, aramid, and carbon fibers were used to create the plain-woven cloth. The behavior of many FMLs was examined using X-ray computed tomography, damaged samples, and graph curve patterns. Fig. 7A displays cross-sectional images of the failed samples, and Fig. 7B(a - d) displays comparison of force‒time, deflection‒time, force‒displacement, and work-time curves of the hybrid AFE and AFB FML laminates. Because PVB-based FMLs have better energy absorption qualities, they performed better in impact tests. In general, the LVI performances of the FMLs reinforced with aramid/jute and PVB were superior.

(Reproduced with permission from John Wiley and Sons License Number – 5642381115393).

The effects of stacking arrangement on the LVI mechanisms of failure and energy dissipation of hybrid laminates made of equal-density carbon and aluminum fibers were investigated by Zhang et al. [27]. The results showed that aluminum layer plastic deformation was the main contributor to energy loss. Aluminum layers stacked on the outside surface may maximize the absorbed impact energy and lessen the tendency of the component layers to fracture. The specimens that contained more layers of carbon fiber reinforced polymer (CFRP) piled on the surface demonstrated more prominent fracture behavior in the constituent layers, resulting in larger areas of delamination. This resulted in increased energy dissipation due to the cracking and delamination of the CFRPs.

**Fig. 8:**
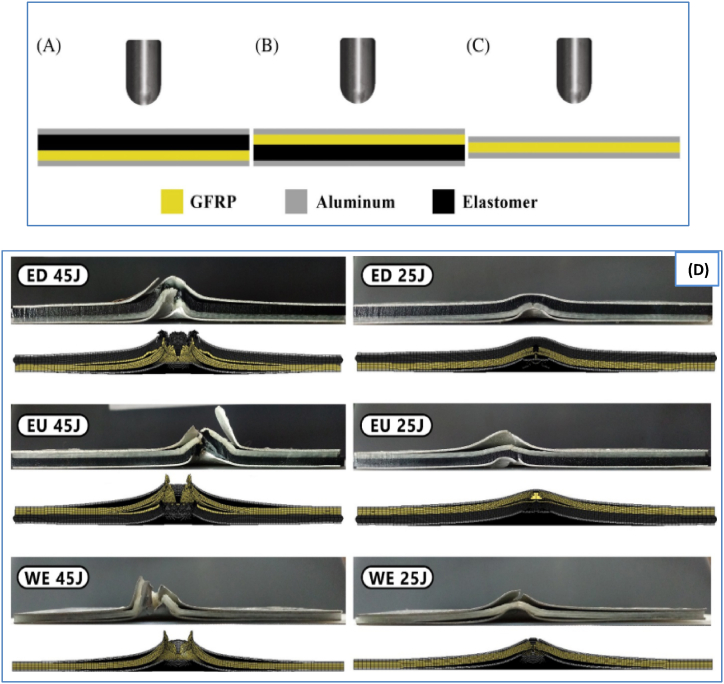
Specimen configurations based on placing the elastomer, (A) the elastomer up (EU), (B) the elastomer down (ED), and (C) without the elastomer (WE), and (D) comparison of sample cross-sections from experimental tests and numerical simulations [26].

Zarezadeh-Mehrizi et al. [26] examined the presence of elastomers on FMLs subjected to LVI loads. Traditional FMLs with layers of Al 6061-T6 and glass/epoxy composite plies were added, and their addition affected the structure's behavior during low-velocity indentation. The addition of an elastomeric layer on the rear surface of the composite layer improved the structural toughness, deformation capacity, and specific energy absorption. Additionally, this modification led to a reduction in damage and the maximum applied load. The configuration of the added elastomer and a comparison of the experimental and numerical failure results are shown in Fig. 8A-D. The FML composite was made using aluminum alloy1060 and glass/carbon fibers fabricated by vacuum bag laminating [28]. This research examined the quasistatic mechanical characteristics and impact-induced dynamic behavior of two distinct fiber laminates. These properties were investigated through tensile tests, 3-point bending tests, and LV impact experiments. The results indicate that GLARE has a greater maximum load and bending strength than CARALL. Bending failure begins at the lower interface of the FRP and spreads to the entire laminate before failure at the lower aluminum alloy layer. Additionally, in regard to bending, GLARE outperforms CARALL in terms of energy absorption.

The LVI response of aluminum-based FMLs was enhanced with glass and carbon fibers in a polymer composite [29]. In connection to various energy levels, the impact of fiber alignments as well as the load‒time curve, damage depth, and area were studied and analyzed. The fiber-metal laminate damage mechanism is quite intricate. Compared to laminates constructed with glass fibers, carbon fiber-reinforced FML composite laminates exhibited a greater inclination to perforate. In contrast to carbon fiber-based laminates, glass fiber-based FML composites absorb energy largely through plastic deformation as they begin the process and spread of delamination.

Carrillo & Cantwell [30] analyzed the mechanical characteristics of a novel self-reinforced polypropylene (SRPP) FML composite and thermoplastic FML with aluminum alloy. The purpose of this study was to examine the effects of varying impact energy levels on the development of damage in FML plates. To achieve this goal, a series of impact tests were conducted. The impacted composite laminates show significant energy absorption, with weakening of the aluminum sheet and cracking of the composite ply as signs of damage. Excellent interfacial adhesion is achieved by bonding the aluminum-SRPP with a modified polypropylene film.

Zhang et al. [31] created a GLARE laminate with a 3/2 configuration by incorporating modified epoxy resins with MWCNTs. They employed a combination of sonication and mechanical stirring to confirm the even distribution of MWCNTs in the epoxy resin. The impact of multiwalled carbon nanotubes (MWCNTs) on the flexural characteristics and impact resistance of GLAREs was examined by conducting bending tests and drop weight impact loading. At elevated levels of impact energy, predominant failure modes, including plastic deformation and tearing of the aluminum sheet, in addition to the breakage of fibers and the matrix, were observed. In contrast, secondary failure modes, MCI (metal composite interfaces), composite layers, and the absorption of energy by the fiber and matrix, were noted at lower impact levels.

Asaee et al. [32] examined the LVI response of 3D fiber metal laminates (3DFMLs) reinforced with graphene nanoplates (GNPs). The resin that is utilized to mate the two major components of the 3DFMLs is strategically reinforced using two types of GNPs: amino-functionalized GNP (NH2-G) and pristine GNP (PG). The results are compared to those of the baseline specimens at three different weight percentages of GNP. The findings show that 1 wt% NH2-G is the ideal content to produce the maximum improvement in the composite's resistance to impact while minimizing the level of damage to the 3DFMLs.

### 2.2 Titanium metal-based FMLs

The structural damage caused by impact loading in fiber metal laminates (FMLs) largely made of titanium [33]. The primary aims of this research were to quantitatively measure the level of internal damage in the in-plane orientation and to evaluate the influence of titanium face sheets on the damage incurred by glass fiber reinforced polymer (GFRP) cores. They observed two kinds of impact responses and damage in Ti/GFRP FML composite laminates. First, after impact loading, the rear side is still intact, and the GFRP layer exhibits a minor amount of interlaminar delamination. Second, at a particular threshold impact energy, a crack is initiated in the titanium metal sheet on the unaffected side, interlaminar delamination in the GFRP layer spreads, and the load response changes at the time of this fracture. Additionally, the researchers conducted a comparison between finite element analysis and comprehensive modeling to assess the impact reactions and damage and subsequently contrasted these findings with experimental data.

Jakubczak et al. [34] studied the effect of the MVF of FML on low-velocity impact loading on new hybrid Ti-CFRP laminates. There are four different forms of hybrid Ti-CFRP laminates with constant are type A: (Ti/0/0/90/90/0/0/Ti/0/0/90/90/0/0/Ti); type B: (Ti/0/0/90/90/0/90/90/0/90/90/0/0/Ti); type C: (Ti/0/90/0/Ti/0/90/90/0/Ti/0/90/0/Ti); type D: (Ti/0/0/90/90/Ti/90/90/0/0/Ti). No significant variations were detected in the maximum contact force, contact time, or damage range. However, it was shown that the energy accumulation capability tended to be limited with an increase in the volume fraction of metal, particularly in the region of lower impact energies. Under low velocity impact conditions, the impact of the composite core thickness on a 2/1 (metal/composite/metal) FML based on titanium alloy was examined by Nassier et al. [35]. It is demonstrated that larger impact force and absorbed energy values are obtained when the composite core's thickness increases.

Sun et al. [36] studied the low-velocity impact response of titanium-based carbon-fibre/epoxy laminates (Ti-FML). The experimental study was conducted with impact energy ranging from 16.9 J to 91.9 J. Three separate damage mechanisms were discovered based on impact energy levels.

Sharma and Velmurugan [37] determined analytically, the LVI behavior and energy absorption processes of FMLs at various impact energy levels prior to the first composite failure. Glass fiber/epoxy layers and titanium alloy Ti-6Al-4V sheets make up four FML layups that reveal the same overall metal layer thickness. Extending their research, Sharma and Velmurugan [38] by conducting the low velocity impact test on Ti-FML laminate and determined that FML with the outermost metallic layers has a higher lateral spreading and interlayer delamination opening than FML with more metallic layers.

Jakubczak and Bienias [39], investigate the damage mechanism of a hybrid titanium carbon laminate, a complete fractography of laminate breakdown was undertaken. The fiber orientation in HTCL has a considerable effect on damage propagation. Reducing interlayers with crossing fibers leads to cracks in titanium's lower layer due to restricted energy absorption by delamination at interfaces.

Kazemi et al. [40] examined the low-velocity impact (LVI) properties of novel thermoplastic (TP) HTCLs at different energy levels. Ti-6Al-4 V sheets, carbon fabrics, and UHMWPE fabrics are utilized to create various laminates with varying fiber types, metal volume fractions, and lamination layups. The laminates are manufactured at room temperature utilizing a low-cost resin infusion process involving a unique liquid thermoplastic methyl methacrylate resin.

### 2.3 Magnesium-based FML

In a research investigation, Asaee et al. [41] fabricated an FML composite structure by including a 3D glass fabric as an intermediate layer between two thin sheets of magnesium. In particular, experimental and computational research has been performed on the LVI response and failure mechanisms of novel FML composites. Moreover, to improve the understanding of the response of FML, a comparative examination is carried out between its efficacy and that of traditional FMLs, which are produced using separate layers of biaxial woven textiles instead of 3D glass fabric. The response of this innovative FML is compared to that of woven fiber FMLs with varying layers, as well as magnesium plates. The results show that FMLs generated from 3D fabrics have superior impact absorption capabilities.

Asaee & Taheri [42] examined the effects of stacking order on the LVI response of an FML composite made of magnesium alloy metal and a real 3D fiberglass fabric using both experimental and computational methods (3DFGF). Four alternative FML configurations and two different 3DFGF thicknesses are considered. The results indicated that the FML-2/2 design layout demonstrated the greatest impact strength when considering cost considerations. In terms of weight, both the FML-2/3 and FML-2/2 design layouts demonstrated similarly impressive impact capacities. Overall, the comparison of the results indicated that both the FML-2/2 and FML-2/3 configurations delivered superior performance across all perspectives.

A significant analytical model is created by Asaee and Taheri [43] to predict the low-velocity impact responses and indentation of 3D fiber metal laminates (3DFML). The impact-induced energy into different 3DFML topologies is considered to dissipate by bending, indentation, and shear contact mechanisms using an energy balance technique.

Deng et al. [44] evaluated the mechanical properties and responsiveness of magnesium-based FML to LVI. The objective of this study was to evaluate the impact of adding MWCNTs (multiwall carbon nanotubes) and GO (graphene oxide). The results showed that, due to their increased stiffness, FMLs reinforced with both GO and WMCNTs showed a greater peak impact load. Additionally, FMLs reinforced with WMCNTs showed a larger region of damage, which resulted in a greater ability to absorb energy during impact.

### 2.4 Steel-based FMLs

**Fig. 9:**
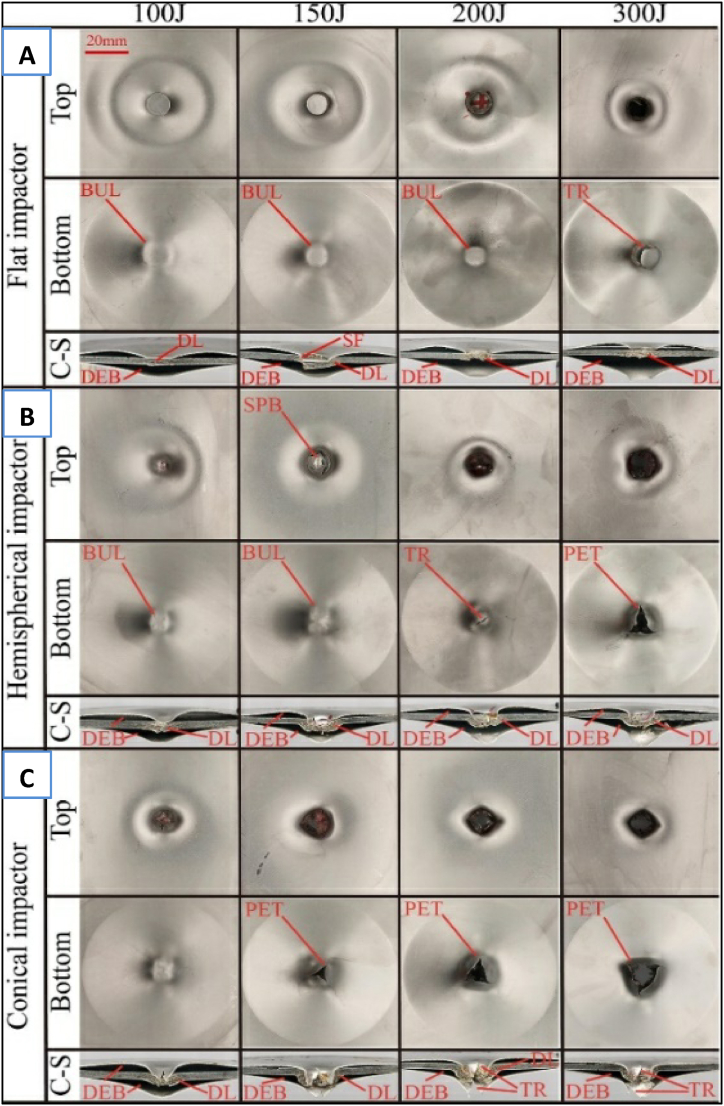
Damages and failures on the top, bottom, and cross-sectional views of FML laminates under different impactor heads A) Flat impactor, B) Hemispherical impactor, C) Conical impactor [45]. (Reproduced with permission from Elsevier License Number – 5604010520142).

Using a drop hammer, Pang et al. [45] examined the LVI response of sandwich FML composite laminates made of steel and basalt fiber with different lay-up topologies. The results indicate that the arrangement of the layers, impact energy level, and impact hammer head shapes all have a notable impact on the behavior of sandwich laminates during impact. The damage and failure modes of the FML laminates with different impactor heads are shown in Fig. 9A-C. When the hammer head is sharper, the peak impact load decreases, and the displacement increases. Under the force of the flat head hammer striking the FML laminate, there is an evident shear failure. When subjected to impact by hemispherical and conical heads, the fractures observed resemble petal-like patterns due to tensile tear failure.

Wei et al. [46] performed a comparison analysis using a drop-weight impact test between two configurations of carbon fiber/ultrathin stainless-steel strip fiber metal laminates (CUSFMLs) with different metal volume contents (35.3 % and 18.2 %). Through experimental and finite element method (FEM) analyses, the impact resistance of CUSFML was evaluated, and the impact response and impact of the metal volume content were investigated. The comparison of two CFRP composite laminates and CUSFML laminates is made easier by the damage inflicted on the laminates. Among the notable considerations were the bearing capacity, impact deflection, failure modes, and energy dissipation. The results indicate that for low-velocity impacts, CUSFML outperforms CFRP composite laminates in terms of impact resistance.

Lee et al. [47] examines the impact behavior of these FMLs, (Carbon FMLs and Glass FMLs) were made by autoclave molding with high-stiffness steel, carbon prepreg, and glass prepreg. Drop-weight impact experiments were used to cause the FMLs to fracture, as well as to examine the behavior of the various laminate sequences in terms of both crack initiation and propagation. The results indicate that the created FMLs are stronger and stiffer than standard FMLs. Their impact strengths increased while weights fell.

The effect of metal-composite debonding on free surface stresses, deformation profiles, and low-velocity impact response was examined by Pärnänen et al. [48]. Focused on type 2/1 fiber metal laminate specimens made of carbon fiber epoxy layers and stainless steel under quasi-static indentation loadings and drop-weight impact. The findings indicated that the slope of the contact force–deflection curve decreases when the force increases due to debonding, whether it be initial debonding or debonding that developed during loading.

### 2.5 Hybrid magnesium/aluminum-based FMLs

Two FMLs were tested for their low-velocity impact reactions: one used magnesium as a metal constituent, the other used AA2024-T3, and both used an SRPP composite [49]. The main objectives of this study were to identify the impact qualities of FMLs and explain the variations in their impact responses. The findings of the study revealed that the Al-FML showed superior resistance to perforation and a greater capacity to withstand energy impact than did the Mg-FML. A summary of the results of low-velocity impact testing on FML laminates is provided in Table 1.

**Table 1:** Overview of studies investigating low-velocity impact behavior in FMLs.

Materials	Metal	Matrix	Type of Fibers	The Major Interpretation in studied	Reference
FML	AA6061-T6	Polypropylene	Glass fiber prepregs	LV impact tests compared surfaces modified with nitrogen plasma treatment and phosphoric acid anodization on aluminum alloy sheets to Al/Gf/PP laminates.	Lin et al. [15]
FML	Al 5052-H32	Polypropylene	Aramid fabric	The LV impact behavior of thermoplastic FMLs, considering different aramid fiber configurations.	Carrillo et al. [21]
FML	AA2024-T3	Polyetherketone-ketone	S-glass fiber	strain rate affects the ability of FMLs made of aluminum to resist perforation in a variety of stacking configurations.	Nassir et al. [24]
FML	Al 6061-T6	Epoxy	Woven glass fibers	The effects of elastomers on FML subjected to LVI loading.	Zarezadeh-mehrizi et al. [26]
FML-2/1	AA2024-T3	Epoxy Resin	UD- carbon and glass fiber	LV impact response of aluminum-based FML enhanced with glass and carbon fibers in a polymer composite.	Jakubczak et al. [29]
3DFML	AZ31B Magnesium alloy	Epoxy	3D fiberglass fabric	The LVI response of an FML is affected by the arrangement of its stacking sequence.	Asaee & Taheri [42]
FML	ZK61m Magnesium alloy	Epoxy	E-glass fibers	The mechanical characteristics and LVI behavior of FML composite are impacted by the inclusion of graphene oxide (GO) and MWCNTs.	Deng et al. [44]
FML	Magnesium alloy, and AA2024-T3	Polypropylene	Self-reinforced Polypropylene	Impact behavior was examined in two FMLs: one with an aluminum base and the other with a magnesium base and self-reinforced polypropylene.	Múgica et al. [49]
CARALL	Aluminum alloy	Epoxy	UD carbon fiber	The response of CARALL to LV impact based on experimental test and numerical analysis. CARALL response a strong resistance to dynamic loading.	Jakubczak et al. [50]
HTCL, CARALL	Titanium, Aluminum alloy	Epoxy	UD carbon fiber	The LV impact response was analyzed for laminates made of CFRP, CLARE, and Ti-CFRP.	Jakubczak et al. [51]
FML-2/1	Stainless steel	Epoxy	UD carbon fiber	The influence of metal-composite debonding on a FML comprised of SS and carbon fiber layers with regard to its LVI response.	Pärnänen et al. [48]
FML	Mild steel	Epoxy	Glass and carbon fiber	Investigate the LVI behavior of steel plate-based FMLs composite according to various stacking orders of glass and carbon prepreg layers.	Lee et al. [47]
FML	AA2024-T3	Epoxy	Unidirectional carbon fiber	Analyze the LVI behavior of FMLs composite laminate composed of AA and CFRP, considering multiple impacts with equal total energy.	Yao et al. [52]
BFML	AA 2A12-T4	Epoxy	Basalt fiber	Comparing the impact response of BFRP plates with that of BFML plates subjected to drop-weight loading.	Zhang et al. [53]
FML	AA2024-T3	Epoxy	UD- carbon fiber	Comparing hybrid CFRP-elastomer-metal laminates (HyCEML) to traditional FML in LVI loading.	Li et al. [54]
GLARE	AA2024-T3	Epoxy Resin	UD glass fiber	Compared the LV response of the virgin GLARE and Nanomodified GLARE(Nylon6,6).	Zarei et al. [55]
CARALL-3/2	AA1050	Epoxy Resin	Carbon fiber	Varying the stacking order of the carbon fiber ply in CARALL grade and compared the LV impact response.	Shi et al. [56]
GLARE 3-2/1	AA2024-T3	Epoxy Resin	UD E-glass fiber	Investigates the impact of the indenter's nose head form on energy absorption and failure mechanisms in low-velocity (LV) impacts.	Ahmadi et al. [57]
FML	AA2024-T3	Epoxy	Woven E-glass fibers	Effect of nanosilica inclusion in the epoxy resin matrix for FML tested in LV impact loading.	Vijayan et al. [58]
CARALL	AA6063-T5	Adhesive FM 300	Carbon fiber prepreg	Evaluate the response and damaging process of CARALL in the context of off-center collisions, simulations and tests are used.	Lu et al. [59]

## 3 High-velocity impact loading testing on FML

High-velocity loadings can be broadly categorized into internal, transitional, exterior, and terminal loadings [60]. The term "terminal ballistics" describes the study of how a projectile interacts with its target. Ballistic impact loading typically involves a low-mass HVI brought on by a launching source. In addition to the qualities of the ballistic material, several other factors could have a significant impact on how well the target material resists ballistic attacks [61,62]. These factors include the impact characteristics, such as the speed, angle of incidence, projectile shape, and boundary conditions of the specimen, as well as the internal and external parameters of the composites and the thickness of the specimen [63].

**Fig. 10:**
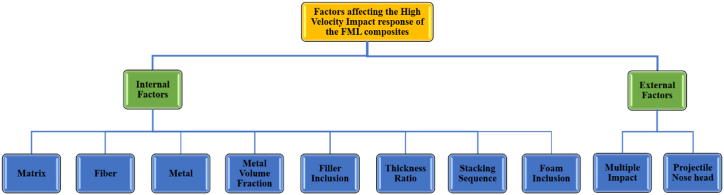
Factors influencing the High-Velocity Impact Behavior of FML Composites.

A projectile is shot via a barrel by a gas gun mechanism to achieve a certain velocity. Several variables, including impact velocity, boundary conditions, projectile shape and mass, material stiffness, strength, and toughness, all influence HVI damage in FML composites, as shown in Fig. 10. Li et al. [64] investigated the dynamic response of GLARE FMLs to the impact of a closed-cell aluminum foam projectile by studying various layup angles with identical layer thicknesses. They identified active failure modes and explored energy absorption mechanisms. Thicker FMLs exhibit reduced damage and deflection. Oblique layups show deformation patterns similar to those of orthogonal layups. The finite element simulations match the experimental results well, especially regarding the deflection over time. The authors also compared the maximum and permanent deflections under different impulses. The energy dissipation ratio of the metal foam projectile is initially the highest but decreases with increasing impulse. The debonding failure energy dissipation decreases with increasing bonding strength.

Zhu et al. [65] investigated the effect of titanium skin on energy dissipation using ballistic experiments on a bimetal fiber metal laminate (FML) made up of Ti-6Al-4 V (TC4) skins and orthogonally woven Kevlar-129 fabric soaked with E54 epoxy resin. They developed a numerical model based on stress wave theory to investigate the energy dissipation mechanism during high-velocity ballistic impact. The numerical results show a considerable and rapid decrease in ballistic velocity with increasing initial incidence velocity in completely inelastic collisions, which is an important characteristic of FMLs after high-velocity impact. Zhang et al. [66] conducted an investigation to improve projectile penetration resistance while maintaining load-bearing and blast mitigation capabilities. They suggested a multifunctional sandwich plate with UHMWPE fiber metal laminate skins and an aluminum honeycomb core. They used a combination of experimental and numerical techniques to evaluate ballistic performance, investigate penetration processes, and understand the underlying mechanics of the innovative sandwich design. Furthermore, the performance of the sandwich plate under three-point bending and impulsive shock loading was evaluated for multifunctional applications. The validation of finite element models against experimental data permitted research into the underlying mechanisms and the impact of structural configurations.

Madika and Syahrial [67] studied the effect of TiC nanoparticles on anti-ballistic materials made from aluminum/Kevlar fiber composite laminates and aluminum/carbon fiber composite laminates. They used three different aluminum alloys (Al-2024, Al-5052, and Al-7075) and two fiber types (kevlar and carbon), which were divided into three categories: Kevlar fibers, TiC nanoparticle-impregnated Kevlar fibers, and carbon fibers. This process generated nine unique composite laminates. Their findings demonstrated that kevlar fibers significantly boosted the yield strength and ultimate tensile strength of composite laminates compared to those of carbon fibers, while carbon fibers had a greater impact on the modulus of elasticity. Among the aluminum varieties, Al-2024 has the best mechanical properties for composite laminates. However, impregnating Kevlar fibers with TiC nanoparticles did not improve the laminates' anti-ballistic properties.

Mansoori et al. [68] investigated the ballistic impact performance of an FML based on aluminum 2024-T3 layers and ultra-high molecular weight polyethylene (UHMWPE) fiber composite is examined using numerical parametric analysis. It has been studied how different parameters, including metal thickness, composite core thickness, metal volume fraction (MVF), and lay-up sequence, affect the samples' ballistic limit velocity, absorbed energy, and specific perforation energy (SPE). The findings indicate that while increasing the thickness of metal layers lowers SPE, increasing the number of composite layers raises SPE. However, the growth of SPE slows down as the core thickness increases. Ballistic velocity and absorbed energy are not significantly affected by changes in the lay-up sequence.

Gao et al. [69] investigated the ballistic and delamination mechanisms of monolithic CFRPs and Al/CFRP/Al composite laminates during high-velocity impact. The carbon/epoxy laminates had a stacking sequence of [−45/90/45/0]. The results showed that the ballistic limit of Al/CFRP/Al laminates of comparable thickness outperformed that of monolithic CFRP laminates, demonstrating greater ballistic performance. Energy absorption was bilinear as impact energy increased, with Al/CFRP/Al laminates absorbing more energy on average than monolithic CFRP laminates. Furthermore, the damage area induced by ball projectile contact on monolithic CFRP laminates was greater than that on Al/CFRP/Al laminates. The distribution and shape of the delamination area were affected by the ply angle and position along the thickness direction.

### 3.1 Effect on the metal volume fraction (MVF)

**Fig. 11:**
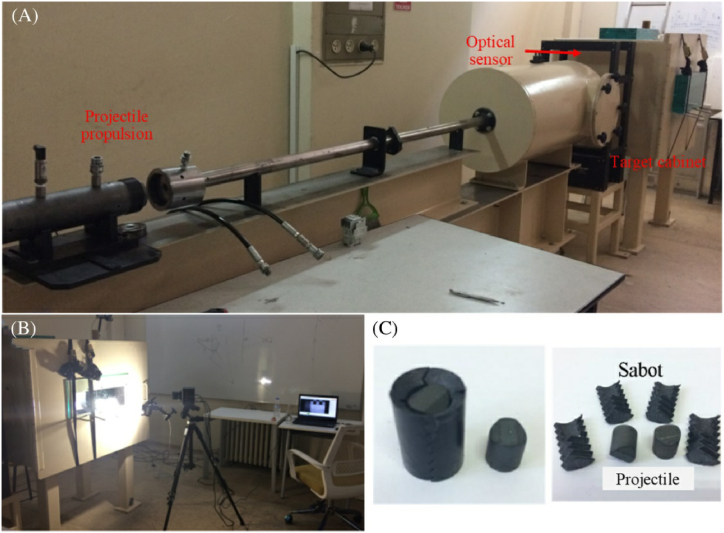
High-velocity impact testing setup: a) gas gun system, b) high-speed camera, and c) projectile [70]. (Reproduced with permission from John Wiley and Sons License Number – 5581341121679).

The effects of fiber orientation, MVF, and the number of layers in FMLs were examined by Kosedag et al. [70]. AA6061 T6 and an aramid fiber reinforced composite were used to make the ARALL. Four distinct varieties of FMLs were generated by varying the metal volume fraction using a hot press and vacuum bag molding. A ballistic impact test was conducted by means of a single-stage gas-gun system, as depicted in Fig. 11a - c. The impact response of the FML composite laminates improved with increasing MVF, and it was revealed that the metal layer that first contacted the projectile had greater impact resistance than did the polymer matrix composite. some.

### 3.2 Effect on filler modification

The most important factor is that a lot of research was done on filler modification using nano and micro fillers in matrix systems. Shahjouei et al. [71] concentrated on the ballistic impact response of AA 2024-T3 sheets and core-reinforced sandwich panels with various GNP concentrations. The results of incorporating GNPs into the epoxy resin revealed that the absorption energy of the composite was negatively impacted by both 0.3 wt% GNP and 0.9 wt% GNP in the resin matrix. The optimal concentration of GNP is mostly dependent on the type of matrix fiber and the initial velocity of the impact test.

Rahmani et al. [72] studied the impact behavior of SiO_2_/ZrO_2_ nanoparticles added to an epoxy resin matrix on CARALL during ballistic impact. To create the CARALL specimens, a carbon fiber, epoxy resin matrix composite containing 0, 1, 3, or 5 wt% of both types of nanoparticles was used to bond a fiber in a 0/90/90/0 stacking sequence between two layers of aluminum 2024-T3. They discovered that adding up to 7 wt% more nanoparticles resulted in the formation of nanoparticle aggregation sites and a reduction in their ability to absorb energy. The inclusion of brittle SiO_2_/ZrO_2_ nanofillers in CARALL was shown to be a potential strategy for increasing the energy absorption ability of CARALL. Vijayan et al. [73] studied the impact resistance of FML composite laminates and the effect of reinforcing nanosilica particles in FML laminates. They altered the composition of nanosilica at concentrations of 0, 1, 3, 5, and 7 wt% and carried out high-velocity impact tests using the ASTM 8101 M standard configuration [74]. The experimental findings indicated that the ballistic limit of the nanosilica-reinforced fiber metal laminate (FML) was 14.3 % greater than that of the pure FML specimen. This research implies that adding nanosilica to FML improves its impact resistance.

In another study, Aghamohammadi et al. [75] explored the influence of MWCNTs on both the flexural test and the HVI response of basalt-FML. The results of the study demonstrate that the incorporation of multiwalled carbon nanotubes (MWCNTs), including aluminum sheets, basalt fibers, and epoxy coatings, had a significant impact on the bonding between the composite layers and the interfaces. The observed increase in adhesion resulted in a significant increase in the flexural characteristics of the MWCNTs incorporated within the fiber metal laminate (FML) framework.

Haro et al. [76] developed hybrid composite laminates using woven Kevlar fiber fabric, epoxy, and AA 5086 aluminum sheets, and they studied the impact of adding micro and nano-fillers to the fiber on the hybrid laminates' ballistic performance. This study included a variety of micro and nano-fillers, including potato flour, aluminum, gamma alumina, silicon carbide, and colloidal silica powders. Under ballistic impact, the energy absorption of hybrid composite laminates with different nano-fillers was measured and contrasted with laminates without nano-filler impregnation.

### 3.3 Effect on matrix materials

The effect of self-reinforced polypropylene (SRPP) and glass reinforced polypropylene (GFPP) are compared by Abdullah & Cantwell [77], focused on investigating the capacity of thermoplastic FMLs to withstand HVI loading. They concluded that the SRPP fiber metal laminates have a slightly lower resistance to penetration than the FML based on the GFPP composite. Additionally, the FML with 2024-T3 has a greater strength than that with 2024-O aluminum alloy sheets. Plastically deforming the aluminum sheet and composite plies consumed much of the energy visible in the longitudinal sectional-cut assessment of the failed specimen.

Ge et al. [78] investigated the impact of altering the quantity of adhesive on the ballistic performance of aluminum/UHMWPE fiber laminates. A series of ballistic impact experiments were performed across a velocity range spanning from 140 to 380 m/s. The energy absorption behavior of the FMLs was hardly affected by the quantity of the MCI adhesive. In the context of evaluating the energy absorption characteristics of three distinct types of fiber metal laminate (FML) specimens, specifically those with 2/1 and 3/2 ratios and varying quantities of adhesive, it was noted that under identical impact velocities, the specimen featuring a moderate adhesive quantity consistently demonstrated the lowest energy absorption capacity. This phenomenon was ascribed to the increased strength of the MCI, which effectively hindered the occurrence of significant debonding.

### 3.4 Effect on thickness ratio

Using a blunt cylinder bullet and a helium gas pistol, Ahmadi et al. [79] studied GLARE-2/1 laminates of different thicknesses at high velocities. It is demonstrated that as the target's total thickness increases, so does the limit velocity or ballistic limit. This means that the target absorbs more energy during perforation if there are additional layers of glass or epoxy.

Ahmadi et al. [80] performed a comprehensive study on the ballistic impact behavior of GLARE laminates. They utilized experimental, analytical, and numerical methods, examining varying thickness ratios of GLARE-3 specimens. The study revealed that the failure pattern of the aluminum sheet on the back side formed a petal shape almost identical to a square shape, aligning with the shape of the blunt head of the projectile. Interestingly, despite significant delamination between the glass/epoxy plates, no debonding occurred between the metal sheets and composite plates. The findings from the analysis revealed that the main mechanism responsible for energy absorption (82%–94 %) was associated with the overall deformation of the aluminum sheets.

Yaghoubi and Liaw [81], conduct an experimental analysis on GLARE 5 (FML) plates, measuring 152.4 mm by 101.6 mm and varying in thickness from 1.12 mm to 4.37 mm, were impact by a bullet-shaped projectile of 0.22 caliber fired from a high-speed gas pistol. The bullet velocity was measured along its ballistic trajectory using a high-speed camera. Both destructive mechanical sectioning and non-destructive ultrasonic sectioning methods were used to assess the post-impact damage characteristics. While the mechanical cross-sectioning technique revealed more data about the damage, the ultrasonic C-scan could only provide the contour of the complete damage area.

**Fig. 12:**
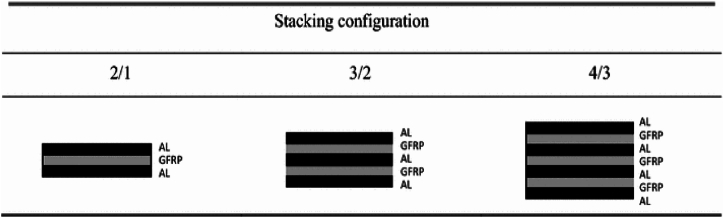
Stacking configuration of the FML sample [82].

Kaboglu et al. [82] examined the performance of fiber metal laminates (FMLs) under projectile impact conditions. Specifically, researchers have investigated the effect of altering the number of aluminum and glass fiber reinforced polymer (GFRP) layers on the impact behavior of FMLs. The flexural properties of the different FML configurations, as shown in Fig. 12, were initially assessed through four-point flexural bending tests. After that, FMLs were subjected to high-velocity impact loading to study their deformation behavior and penetration resistance. Research has shown that augmenting the quantity of metallic and composite layers offers benefits in terms of energy absorption. These advantages are primarily attributed to various mechanisms, including plastic ripening, petalling, and fracture of the aluminum layers, as well as delamination, fracture of the GFRP layers, and fiber breaking.

### 3.5 Effect on fibers

The failure of FML composite laminates was investigated by Li et al. [83]. To strengthen the aluminum sheets, 3 forms of woven fibers, S2-glass, basalt, and Kevlar-29, were used. The FML panels were subjected to impact velocities ranging from 20.6 m/s to 42.8 m/s for testing. By altering the impact energy, different failure modes of FML were achieved. The findings of the study revealed a clear and positive relationship between the velocity of impact and the overall deformation observed, specifically in relation to the penetration of the fiber metal laminate panels. However, after penetration, there is a clear reduction in the overall distortion. Vlot [84] analyzed the impact response of fiber metal laminates (FMLs) under various loading conditions, including static, LVI, and HVI loading. These experiments were performed using a hemispherical steel indentor with a radius of 7.5 mm. Compared with glass and carbon fiber composite materials, FML sustains significantly less severe damage. The delaminated zone of the failed composite specimens was identified by C-ray scanning. The tiniest circle surrounding the delaminated area is used to define the damage width. To measure the extent of the failure damage, the FML outer layers of aluminum were peeled off. Because of the plastic deformation of the dent, which reflects ultrasonic waves, these specimens cannot be C-scanned.

### 3.6 Effect on the stacking sequence

**Fig. 13:**
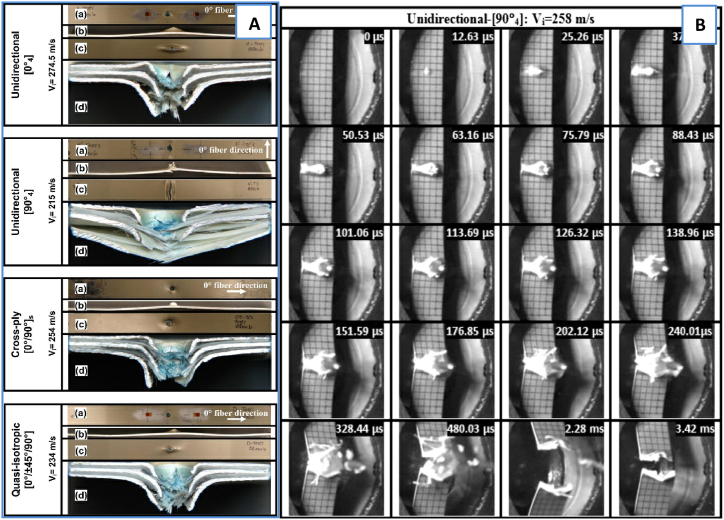
A) Damage induced in the FML impacted at various incident velocities. B) Impact pictures of a high-speed camera of a projectile on GLARE 5 FML [85] (Reproduced with permission from Elsevier License Number - 5604011394966).

Investigating how lay-up orientation affects FML impact performance might be more fascinating. A comprehensive investigation of the behavior of GLARE 5 FML beams subjected to ballistic impact was conducted by Seyed Yaghoubi & Liaw [85]. This investigation involved both experimental and numerical analyses. They considered different stacking sequences, including UD, cross-ply, and quasi-isotropic configurations. Ballistic impact tests were performed using a high-speed camera and gas gun setup. Using high-speed video footage and optical imaging, the sudden damage in the specimens was determined. The results showed that GLARE 5 (3/2) generated damage that resembled that of a monolithic aluminum alloy and a composite laminate formed of polymers under various stacking sequences. The prepreg lay-up orientation, interfacial delamination/debonding intensity, and impact energy in the FML beams were expected to decrease the most due to aluminum layer bending and stretching. The damage in the FML laminate is shown in Fig. 13A. Pictures of the high-speed camera are shown in Fig. 13B for various stacking sequences.

Khan and Sharma [86] investigated FML laminates, which are interface failures in metal/GFRP laminates caused by a hemispherical bullet impacting at high velocity. There are three varieties of FMLs, and the failure of the eight-layer (0/90) GFRP laminate is compared to the failures of the other two laminates, which have metal layers in their layup structure: 2/1–0.6 ([A6/0/90/90/0/A6]) and 4/3–0.3 ([A3/0/A3/90]_s_). On one variation of the laminate, the metal layers are positioned on top and bottom, while on the other, extra metal layers are proportionately positioned inside the laminate.

**Fig. 14:**
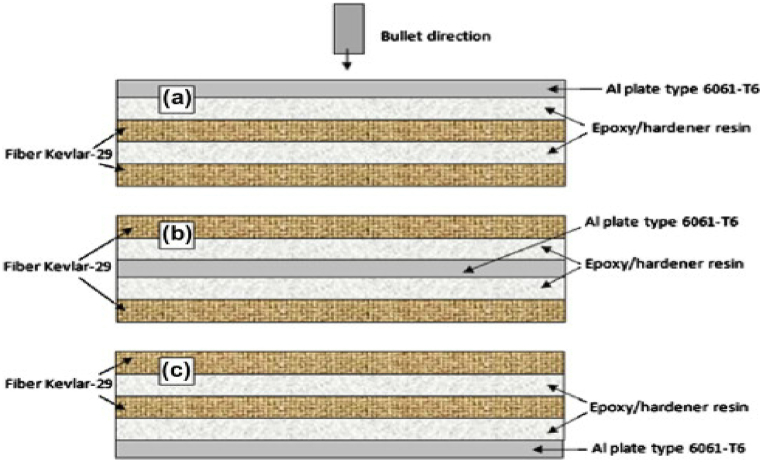
Stacking sequence order of the aluminum plate inside the laminated (a) upper, (b) middle and (c) lower layers. (Reproduced with permission from Elsevier License Number – 5641990456580).

In another study, Ramadhan et al. [87] employed a nitrogen gas cannon to test the HVI response on FML laminates made of Kevlar fiber/AA6061-T6/epoxy resin stacked in various configurations. To completely perforate the target, impact testing was performed using a cylindrical steel projectile at various speeds (180–400 m/s). They also examined the front, center, and back positions of the aluminum plates in the laminated plates of the composite specimen, as shown in Fig. 14a - c. The best arrangement to withstand impact loading, according to the overall results, was the aluminum-back stacking sequence plate. The simulation model effectively predicted the residual velocity of bullets piercing Kevlar/epoxy woven with aluminum laminated plates. Similarly, Sharma et al. [88,89,90] investigates the high velocity impact response of titanium reinforced fiber metal laminate by varying stacking sequences of metal and UDGRFP layers and examined the damage performance of failed FML laminates.

### 3.7 Effect on foam inclusions

**Fig. 15:**
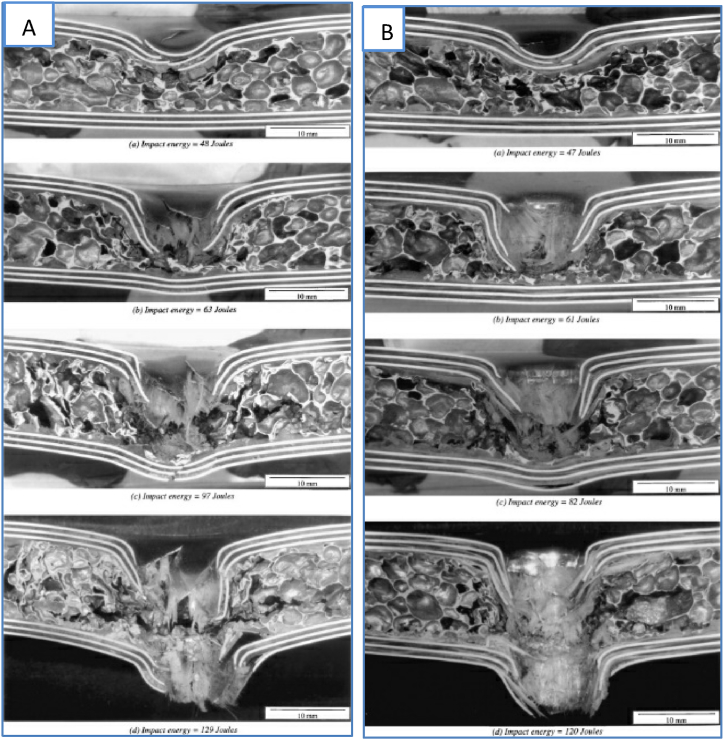
Cross-sectional images of failed FML sandwich laminates: a) UD and b) Woven composite [91]. (Reproduced with permission from Elsevier License Number – 5604020984546).

With respect to reinforcing foam as a core material, the FML response is more effective. The effect of FML aluminum foam sandwich structures with aluminum skins was investigated by Reyes & Cantwell [91]. In high-velocity impact testing, the fibers are divided into unidirectional and woven glass fiber/polypropylene-based systems. Consequently, several alternative failure mechanisms were observed, and the analytical model was utilized to forecast the limit velocity of each laminate. The anticipated velocity limit of the FML laminates with aluminum foam cores was determined using Reid and Wen's model. A sectional view of the failed structures is shown in Fig. 15a, b.

### 3.8 Effect on projectile nose head variation

**Fig. 16:**
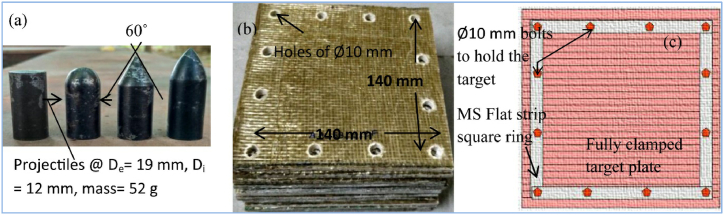
a) Various nose head projectiles, b) GFRP laminates for high-velocity impact, and c) clamping target plates [92]. (Reproduced with permission from Elsevier License Number – 5604010767619).

The shape of the projectile's nose head is a crucial factor that influences the failure morphology of the FML laminate. A composite laminate reinforced with unidirectional glass fibers of various thicknesses was tested under high-velocity impact (HVI) [92]. A 52 g steel projectile was employed in the experiment, with several nose forms, such as conical, flat, ogival, and hemispherical. The projectile was launched with an initial velocity of 300 m/s. Delamination was the primary cause of target failure, and the magnitude of the damage experienced by the projectile exhibited an upward trend when its nose shape underwent a transition from conical to flat, ogive, and hemispherical configurations. Target damage was greatest when the projectile hit perpendicularly, and laminated composite damage increased with increasing incidence angle. The configuration of the projectile, laminate and clamping details are shown in Fig. 16a - c.

In a study [93], the authors examined the energy absorption and impact response of an FML composed of AA 2024T3 sheets and a composite fiber constructed of UHMWPE. The failure patterns and impact performances of the composite cores and aluminum sheets of various thicknesses were compared for two different bullet types. In a test using a spherical projectile, the increased thickness of the composite core prevented the projectile from penetrating completely and, at greater thicknesses, minimized damage to the back side of the laminate. The elasticity of the composite core enables it to deform in step with the deformation of the aluminum sheet. In testing using a conical nose head projectile, the aluminum layer failure mechanism included plugging and petaling, and the composite core damage modes included fiber breakage, splitting, and delamination.

Majzoobi et al. [94] mathematically and empirically investigated the failure mechanism, absorbed energy, ballistic limit, and specific perforation energy (SPE) of FML 2/1 and FML 3/2 using one-stage gas cannon and high-velocity impact loading. In addition, three different projectile nose geometries are used to examine the ballistic impact response of FML composite laminates. The findings showed that the conical-nose head projectile had the highest velocity limit and that FML-3/2 had a ballistic limit 75.6 % greater than that of FML-2/1. Compared to FML-2/1, FML-3/2 had SPE values of flat, hemispherical, and cone-shaped nose head projectiles that improved by 72.51 %, 55.26 %, and 89.22 %, respectively. To determine how the strain rate and projectile nose shape affect the ballistic limit of CRALL, Gaur et al. [95] conducted dynamic explicit analyses under high-velocity impact (HVI) conditions. For this purpose, projectiles that were flat, conical, and hemispherical nose heads were used with three different strain rates.

### 3.9 Effect on multiple impacts

As the number of impacts on the laminate increases, the failure of the material expands. The ballistic impact deformation behavior of Ti-FML was investigated by Suresh Kumar et al. [96]. Boron carbide filler particles were added to Ti-FML laminates at weight percentages of 5 % and 10 %. The mechanical characteristics of the Ti/GFRP FML samples were assessed in accordance with ASTM standards. Armor piercing piles (APPs) with velocities ranging from 350 to 450 m/s were used in high-velocity ballistic studies. The depth of penetration of the projectile into the target was calculated.

### 3.10 Effect of the projectile angle of incidence

Chen et al. [97] experimentally studied the effect of varying the projectile angle of incident on GLARE laminates subjected to ballistic impact at normal (0°) and oblique (30° and 45°) angles using a rigid cylindrical projectile. The FMLs underwent ballistic impact testing using a gas gun setup at impact angles of 0°, 30°, and 45°. Sangsefidi et al. [98] investigated how the deformability of projectiles influences the response of FMLs in HV impact experiments. A light gas pistol with rigid, semirigid and deformable projectiles of equal size and mass was utilized. FML-2/1 was made with varying layers of glass/epoxy composite. This study analyzed the mechanisms for energy absorption and determined the ballistic limits and specific perforating energy for each case.

### 3.11 Effect on metal constituents

The metal-adhered layers of the FML composite play a crucial role as the outermost layers, contributing to the ductile strength of the laminate. Researchers employ a variety of metal sheets, including aluminum, titanium, magnesium, and steel. Abdullah & Cantwell [99] studied the ballistic impact response of AA sheet and woven GFRP polypropylene FMLs. The analysis of the failed samples revealed that energy absorption occurred through mechanisms such as fiber fracture, matrix deformation, and delamination within the composite layers. Additionally, metal fracture and plastic distortion and fracture were observed in the aluminum plates. The research findings indicate that 2024-T3 FMLs exhibit greater resistance to perforation than do 2024-O laminates. This can be attributed to the increased capacity of the T3 alloy to absorb energy.

Majzoobi et al. [100] investigated FMLs with three distinct layer configurations, namely, Al/R/Al, Ti/R/Al, and Al/R/Ti arrangements, to determine their energy absorption, velocity limit, and failure mechanism. Each FML specimen has two layers of metal, either titanium 6Al-4 V or aluminum 2024-0(Al), reinforced with E-glass unidirectional fiber and epoxy resin. Both experimental and simulation methods were used to conduct the investigation. The Al/R/Ti specimen exhibited a higher ballistic limit than both the Ti/R/Al and Al/R/Al specimens.

**Fig. 17:**
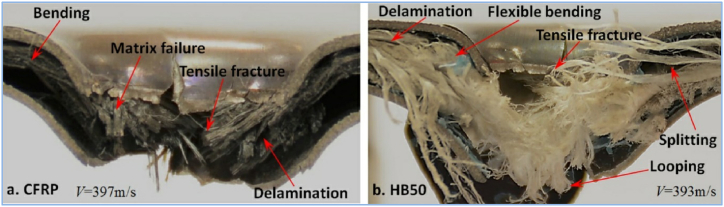
Vicinity of damage morphology of composite layers and metal sheets in FML: a) CFRP and b) HB50 [101]. (Reproduced with permission from Elsevier License Number – 5604020425483).

Li et al. [101] studied how fiber type affects Ti-based FML failure. This study involved two distinct FML configurations consisting of layers of carbon fiber-based Ti/CFRP sandwiched composite laminates. The second FML was made of UHMWPE fiber layers as opposed to the first FML's CFRP layers. A steel sphere hits these FMLs at varying velocities in the range of 100–400 m/s. Based on the results, the Ti/UHMWPE laminate system offers superior ballistic performance to that of the Ti/CFRP combination, especially in the areas of limited velocity and energy absorption. Fig. 17(a, b) presents a cross-sectional perspective of the material, providing insight into the morphology of the failure.

**Fig. 18:**
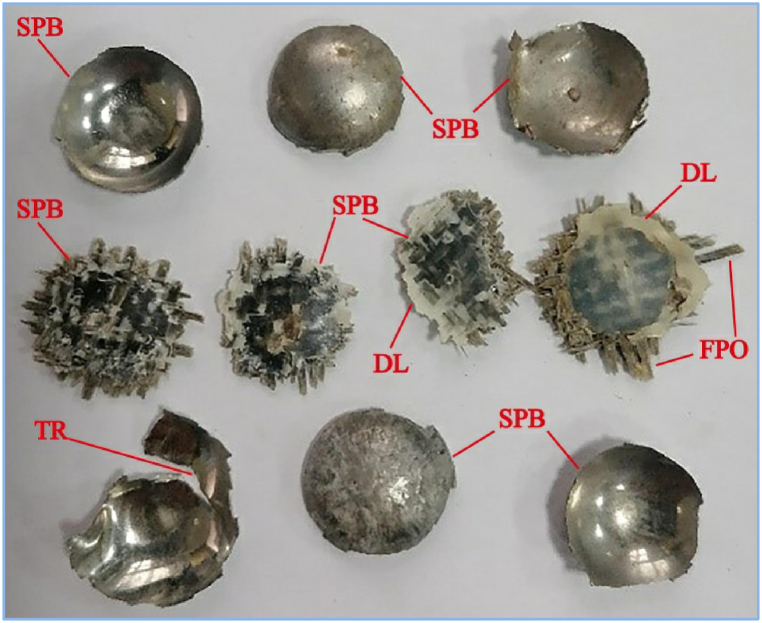
Pieces of the perforated FML structure. SPB: shear plugging block, FPO: fiber pull-out [102]. (Reproduced with permission from Elsevier License Number – 5604020683674).

A basalt fiber/steel laminate was subjected to a ballistic impact test utilizing a steel ball projectile from a single-stage gas cannon [102]. The goal of this analysis is to determine how the laminated surface density, lay-up structure, laminate impact angle, and fiber volume fraction affect the ballistic impact performance. The results indicated that the laminates with different lay-up configurations, specifically FML-3/2, FML-2/1, and FML-4/3, exhibited decreasing limit velocities in the order of high to low during normal penetration. When the projectile perforated the target, the ballistic limit velocity for the 3/2 lay-up laminate decreased with increasing fiber volume fraction, while the SEA increased with increasing laminated surface density. Fig. 18 shows the plugged pieces of the perforated samples. The high-velocity impact loads on the FML laminates are summarized in Table 2.

**Table 2:** Overview of studies investigating high-velocity impact behavior in FMLs.

Materials	Metal	Matrix	Type of Fibers	The major Interpretation in studied	Reference
ARALL	Al6061 T6	Epoxy	Aramid fiber	Studied the influence of stacking order, metal volume percentage, and layer count on FML in ballistic impact.	Kosedag et al. [70]
FML	Al2024-T3	Epoxy	UD E-Glass fiber, carbon fiber	The effect of GNP in FML to HV impact loading response.	Shahjouei et al. [71]
FML – 3/2	AL 1100-O	Epicote 2175	Woven Kevlar, S2 glass fiber, Basalt fiber	The dynamic failure analysis of the three kinds of the woven fiber materials in FML was studied in projectile impact.	Li et al. [83]
FML	AA2024-T3	Polyure- thane	UHMWPE fiber	The HV impact performance of composite core and aluminum sheets of various thicknesses was studied.	Mansoori et al. [93]
Ti-FML	Titanium alloy Ti-6Al-4 V	Epoxy	Woven carbon fabric	The effect of the specific FRP used as a core layer in Titanium-based FMLs when subjected to impacts from steel projectiles.	Li et al. [101]
FML	304 SS	Epoxy	Basalt fiber	Ballistic impact performance of FML is determined by the lay-up structure, FVF, laminated surface density, and impact angle.	Pang et al. [102]
FML	AA 5086-H32	Polyethylene glycol	Kevlar fabrics were	Research on the ballistic response of FML for fillers such as potato flour, silicon carbide, gamma alumina, aluminum, and colloidal silica.	Haro et al. [76]
FML	AA2024-T3	Epoxy	UD glass fiber	The impact response of projectiles is affected by the distribution of metal layers throughout the thickness of FML.	Sharma & Khan [103]
FML	AA2024-T3	Epoxy	Woven basalt fiber	The influence of including nonconductive (NC) materials on the mechanical properties and impact behavior of laminated composites composed of basalt fibers and epoxy-aluminum 2024-T3.	Bahari-Sambran et al. [104]
FML	AL 5005	Epoxy	Woven E glass fiber	Sandwich panels with FML constructed of plain-woven E glass fiber composite plies, aluminum sheets as skins, and aluminum foam as cores demonstrate high velocity impact reactions.	Liu et al. [105]
FML	Ti-6Al-4 V titanium alloy	Epoxy	UD E-glass fiber	The influence of metallic layer distribution over FML thickness on projectile impact reaction and damage.	Sharma et al. [89]
FML	AA2024-T3	Epoxy	Woven glass fiber	The effect of nanosilica reinforced FML in ballistic impact loading and its dynamic response	Vijayan et al. [73]

The interface failure in metal/GFRP laminates caused by a hemispherical bullet impacting at high velocity is the main topic of this paper. Three laminates are examined in the study, and the failure within the eight-layer 0/90 GFRP laminate is contrasted with the failures of the other two laminates, which have metal layers in their layup structure. On a specific type of laminate, the metal layers were positioned on the top and bottom, while on the other, there are extra metal layers symmetrically positioned inside the lamination.

## 4 Future scenarios for the impact loading response of FML

● Hybrid composite laminates can be created using 3D printing. This method can be expanded to produce planned constructions with unique and personalized configurations and compositions. The current focus is on developing FMLs with properties similar to those of commercially available FMLs by utilizing internally and continuously reinforced composites that have been 3D printed [106,107].
● The use of natural fibers in FMLs to make lightweight, robust fabrics is expanding. One suitable application for natural fiber-reinforced FMLs is the construction of car front hoods [108].
● In FML laminates, the fabrication of curved surfaces is a real-time process that is studied under impact loading.
● In the area of oblique impact loading, the response of FML laminates when projectiles impact them at various incident angles is studied by varying the specimen angle.
● The analysis of the impact response varies based on the initial contact surface of the FML, whether it is metal or composite, which is exposed to the projectile's nose head.
● Testing also includes variations in preconditioning states, such as prestress and precompression loads applied to the specimen before impact.
● Key research areas include testing after impact, such as compression after impact, flexural strength after impact, and tensile strength after impact, which are areas of current focus after specimen failure.
● Research on techniques for repairing failed samples is also at an advanced stage. This includes methods such as scarf patching, the choice of materials for patching, and the orientation of the patch composite, among others.
● One potential future scenario involves the development of advanced computational modeling and simulation techniques to accurately predict the impact behavior of FMLs under various loading conditions. These models could incorporate material properties, failure mechanisms, and deformation behavior to optimize the design and configuration of FML structures for enhanced impact resistance. Sophisticated numerical techniques, such as finite element analysis (FEA) and multiscale modeling, could be employed to capture the complex interactions between the metal and composite layers during impact events.
● Another scenario involves the exploration of novel material combinations and architectures for FMLs to improve their impact performance. This could involve the incorporation of high-strength and high-toughness metal alloys, advanced fiber reinforcements (e.g., carbon nanotubes, graphene), and the development of graded or functionally graded material systems. Additionally, the introduction of through-thickness reinforcement or z-pinning techniques could enhance the interlaminar strength and delamination resistance of FMLs under impact loading.
● Furthermore, the integration of self-healing capabilities into FMLs could be a promising future direction. The incorporation of self-healing mechanisms, such as microencapsulated healing agents or shape memory alloys, could enable FMLs to autonomously repair damage caused by impact events, prolong their service life and enhance their overall durability.
● In terms of applications, FMLs with superior impact resistance could be extensively used in various industries, including the aerospace, automotive, and defense sectors. For instance, FMLs could be employed in the construction of aircraft fuselages, armored vehicles, and protective structures, where resistance to ballistic impacts, foreign object damage (FOD), and other high-energy impact events is critical.

The development of lightweight and impact-resistant FMLs could contribute to the broader goals of energy efficiency and sustainability by enabling the design of lighter and more durable structures and reducing fuel consumption and carbon emissions in transportation applications. Future scenarios for the impact loading response of FMLs involve multidisciplinary efforts involving material science, computational modeling, manufacturing techniques, and application-specific design considerations. Continued research and development in this area have the potential to unlock new possibilities for advanced composite materials with enhanced impact resistance, enabling innovative solutions across various industries.

## 5 Conclusion

This comprehensive review has scrutinized recent advancements in understanding the impact response of fiber metal laminates (FMLs) under both low-velocity and high-velocity impact loading scenarios. The unique hybrid construction of FMLs, which combine metallic and composite constituents, exhibits exceptional impact resistance and damage tolerance capabilities.

For low-velocity impacts, this review delved into the intricate damage mechanisms, energy absorption characteristics, and profound influence of various parameters. These include the effects of filler modification, matrix materials, thickness ratios, fiber types, stacking sequences, and foam inclusions on the impact response. The ability of FMLs to dissipate impact energy through multiple mechanisms, such as fiber bridging, delamination, and metal plastic deformation, has been extensively explored.

Regarding high-velocity impact loading, this review critically analyzed the role of FMLs in mitigating perforation and enhancing damage tolerance under scenarios such as foreign object damage and ballistic impacts. The effects of projectile characteristics, including nose shape variations and multiple impacts, as well as the projectile's angle of incidence, have been thoroughly discussed. Additionally, the influence of metal constituents and multilayer configurations on the high-velocity impact response has been highlighted.

This review has emphasized the importance of accurate impact modeling and advanced experimental techniques for validating and refining the understanding of FML impact behavior under both low- and high-velocity loading conditions. Despite the significant progress made, several challenges and future research directions have been identified. These include the development of more robust computational models, the exploration of novel FML configurations and material combinations, and the investigation of multihit and oblique impact scenarios under varying loading rates. Overall, this review has underscored the immense potential of FMLs in applications demanding superior impact resistance and damage tolerance, such as the aerospace, automotive, and defense industries. The findings presented herein contribute to ongoing efforts in developing lightweight, impact-resistant structural components, ultimately advancing the state of the art across various engineering domains.

## CRediT authorship contribution statement

**Vijayan Muniyan:** Writing – review & editing, Writing – original draft, Visualization, Validation, Software, Resources, Methodology, Investigation, Formal analysis, Data curation, Conceptualization. **Vishnu Vijay Kumar:** Writing – review & editing, Visualization, Validation, Supervision, Resources, Investigation, Conceptualization. **Indran Suyambulingam:** Writing – review & editing, Visualization, Validation, Supervision, Software, Methodology, Investigation, Formal analysis, Data curation, Conceptualization. **Suganya Priyadharshini:** Writing – review & editing, Visualization, Validation, Resources, Methodology, Investigation, Conceptualization. **Divya Divakaran:** Writing – review & editing, Visualization, Validation, Methodology, Investigation, Data curation, Conceptualization. **Sanjay Mavinkere Rangappa:** Writing – review & editing, Visualization, Validation, Software, Resources, Methodology, Investigation, Formal analysis, Data curation, Conceptualization. **Suchart Siengchin:** Writing – review & editing, Visualization, Validation, Software, Resources, Methodology, Investigation, Data curation, Conceptualization.

## Data availability

The data that support the findings of this study are available upon request from the corresponding author.

## Funding

The authors express their gratitude to the 10.13039/501100001427All India Council for Technical Education (AICTE), located in New Delhi, India, for providing the research grant through the RPS-NDF scheme (8–24/2018-19).

## Declaration of Competing Interest

The authors declare that they have no known competing financial interests or personal relationships that could have appeared to influence the work reported in this paper.

## References

[1] Arora G., Pathak H. (Jan. 2019). Multi-scale fracture analysis of fibre-reinforced composites. Mater. Today Proc..

[2] Asundi A., Choi A.Y.N. (1997). Fiber metal laminates: an advanced material for future aircraft. J. Mater. Process. Technol..

[3] Liu Y., Zhang R., Liang E.Q., Li D., Chen Y., Zhang J. (2014). A review on development and properties of GLARE, an advanced aircraft material. Appl. Mech. Mater..

[4] Vlot A. (2001).

[5] Vlot A., Vogelesang L.B., De Vries T.J. (1999). Towards application of fibre metal laminates in large aircraft. Aircr. Eng. Aerosp. Technol..

[6] Botelho E.C., Silva R.A., Pardini L.C., Rezende M.C. (2006). A review on the development and properties of continuous fiber/epoxy/aluminum hybrid composites for aircraft structures. Mater. Res..

[7] Sinmazçelik T., Avcu E., Bora M.Ö., Çoban O. (Aug. 2011). A review: fibre metal laminates, background, bonding types and applied test methods. Mater. Des..

[8] Katnam K.B., Da Silva L.F.M., Young T.M. (Aug. 2013). Bonded repair of composite aircraft structures: a review of scientific challenges and opportunities. Prog. Aerosp. Sci..

[9] da Silva L.F.M., Campilho R.D.S.G. (2015). Fatigue and Fracture of Adhesively-Bonded Composite Joints.

[10] Pundhir N., Arora G., Pathak H., Zafar S. (2020). Lecture Notes in Mechanical Engineering.

[11] Vijayan M., Selladurai V., Vijay Kumar V., Balaganesan G., Marimuthu K. (2024). Low-velocity impact response of nano-silica reinforced aluminum/PU/GFRP laminates. Springer Proc. Mater..

[12] Jakubczak P., Bieniaś J., Surowska B. (2017). Hybrid Polymer Composite Materials: Properties and Characterisation.

[13] Sharma A.P., Khan S.H., Kitey R., Parameswaran V. (October 2017). Effect of through thickness metal layer distribution on the low velocity impact response of fiber metal laminates. Polym. Test..

[14] Caprino G., Spataro G., Del Luongo S. (May 2004). Low-velocity impact behaviour of fibreglass-aluminium laminates. Compos. Part A Appl. Sci. Manuf..

[15] Lin Y., Li H., Zhang Z., Tao J. (2021). Low-velocity impact resistance of al/gf/pp laminates with different interface performance. Polymers.

[16] Sisan M.M., Eslami-Farsani R. (Oct. 2019). An experimental study on impact resistance of different layup configuration of fiber metal laminates. Fibers Polym..

[17] Laliberté J.F., Straznicky P.V., Poon C. (2005). Impact damage in fiber metal laminates, part 1: experiment. AIAA J..

[18] Fathi A., Liaghat G., Sabouri H. (2021). An experimental investigation on the effect of incorporating graphene nanoplatelets on the low-velocity impact behavior of fiber metal laminates. Thin-Walled Struct..

[19] Tsartsaris N., Meo M., Dolce F., Polimeno U., Guida M., Marulo F. (2011). Low-velocity impact behavior of fiber metal laminates. J. Compos. Mater..

[20] Bagnoli F., Bernabei M., Figueroa-Gordon D., Irving P.E. (2009). The response of aluminium/GLARE hybrid materials to impact and to in-plane fatigue. Mater. Sci. Eng. A.

[21] Carrillo J.G., Gonzalez-Canche N.G., Flores-Johnson E.A., Cortes P. (Jul. 2019). Low velocity impact response of fibre metal laminates based on aramid fibre reinforced polypropylene. Compos. Struct..

[22] Yarmohammad Tooski M., Alderliesten R.C., Ghajar R., Khalili S.M.R. (2013). Experimental investigation on distance effects in repeated low velocity impact on fiber-metal laminates. Compos. Struct..

[23] Ferrante L., Sarasini F., Tirillò J., Lampani L., Valente T., Gaudenzi P. (2016). Low velocity impact response of basalt-aluminium fibre metal laminates. Mater. Des..

[24] Nassir N.A., Birch R.S., Cantwell W.J., Al Teneiji M., Guan Z.W. (2021). The perforation resistance of aluminum-based thermoplastic FMLs. Appl. Compos. Mater..

[25] Hussain M., Imad A., Nawab Y., Saouab A., Herbelot C., Kanit T. (May 2022). Effect of matrix and hybrid reinforcement on fibre metal laminates under low–velocity impact loading. Compos. Struct..

[26] Zarezadeh-mehrizi M.A., Liaghat G., Ahmadi H., Taherzadeh-Fard A., Khodadadi A. (Apr. 2022). Numerical and experimental investigation of fiber metal laminates with elastomeric layers under low-velocity impact. Polym. Compos..

[27] Zhang F. (Aug. 2022). Comparison of stacking sequence on the low-velocity impact failure mechanisms and energy dissipation characteristics of CFRP/Al hybrid laminates. Polym. Compos..

[28] Deng Y., Wang R., Liang X., Peng J., Wei G. (Nov. 2023). Study on the failure mechanism of 1060-H112 aluminum alloy-carbon/glass fiber laminate. Polym. Compos..

[29] Jakubczak P., Surowska B., Bieniaś J. (Apr. 2016). The comparison of low-velocity impact resistance of aluminum/carbon and glass fiber metal laminates. Polym. Compos..

[30] Carrillo J.G., Cantwell W.J. (2009). Mechanical properties of a novel fiber-metal laminate based on a polypropylene composite. Mech. Mater..

[31] Zhang H., Gn S.W., An J., Xiang Y., Yang J.L. (2014). Impact behaviour of GLAREs with MWCNT modified epoxy resins. Exp. Mech..

[32] Asaee Z., Mohamed M., De Cicco D., Taheri F. (2017). Low-velocity impact response and damage mechanism of 3D fiber-metal laminates reinforced with amino-functionalized graphene nanoplatelets. Int. J. Compos. Mater..

[33] Nakatani H., Kosaka T., Osaka K., Sawada Y. (2011). Damage characterization of titanium/GFRP hybrid laminates subjected to low-velocity impact. Compos. Part A Appl. Sci. Manuf..

[34] Jakubczak P., Bieniaś J., Droździel M., Podolak P., Harmasz A. (2020). The effect of layer thicknesses in hybrid titanium-carbon laminates on low-velocity impact response. Materials.

[35] Nassir N.A., Birch R.S., Cantwell W.J., Guan Z.W. (Sep. 2023). The influence of composite core thickness on the perforation resistance of titanium-based FMLs. Results Mater.

[36] Sun J., Chen W., Luo H., Xie X., Zhang J., Ding C. (Nov. 2024). Low-velocity impact behaviour of titanium-based carbon-fibre/epoxy laminate. Materials.

[37] Sharma A.P., Velmurugan R. (2022). Analytical modelling of low-velocity impact response characterization of titanium and glass fibre reinforced polymer hybrid laminate composites. Thin-Walled Struct..

[38] Sharma A.P., Velmurugan R. (Dec. 2022). Damage and energy absorption characteristics of glass fiber reinforced titanium laminates to low-velocity impact. Mech. Adv. Mater. Struct..

[39] Jakubczak P., Bieniaś J. (2021). The response of hybrid titanium carbon laminates to the low-velocity impact. Eng. Fract. Mech..

[40] Kazemi M.E. (Oct. 2021). Developing thermoplastic hybrid titanium composite laminates (HTCLS) at room temperature: low-velocity impact analyses. Compos. Part A Appl. Sci. Manuf..

[41] Asaee Z., Shadlou S., Taheri F. (2015). Low-velocity impact response of fiberglass/magnesium FMLs with a new 3D fiberglass fabric. Compos. Struct..

[42] Asaee Z., Taheri F. (2016). Experimental and numerical investigation into the influence of stacking sequence on the low-velocity impact response of new 3D FMLs. Compos. Struct..

[43] Asaee Z., Taheri F. (Jan. 2020). A practical analytical model for predicting the low-velocity impact response of 3D-fiber metal laminates. Mech. Adv. Mater. Struct..

[44] Deng Y., Hu A., Wei G., Wang R., Yin Y., Zhou C. (Feb. 2022). Experimental study of the effect of GO/WMCNTs on the mechanical properties and impact response of magnesium-based fiber metal laminates. Polym. Compos..

[45] Pang Y., Yan X., Yao H., Qu J., Wu L. (2022). Experimental study of basalt fiber/steel hybrid laminates under low-velocity impact. Eng. Fract. Mech..

[46] Wei S., Zhang X., Li Y., Wang T., Huang Q., Liu C. (2024). Study of the dynamic response and damage evolution of carbon fiber/ultra-thin stainless-steel strip fiber metal laminates under low-velocity impact. Compos. Struct..

[47] Lee D.W., Park B.J., Park S.Y., Choi C.H., Il Song J. (Apr. 2018). Fabrication of high-stiffness fiber-metal laminates and study of their behavior under low-velocity impact loadings. Compos. Struct..

[48] Pärnänen T., Vänttinen A., Kanerva M., Jokinen J., Saarela O. (2016). The effects of debonding on the low-velocity impact response of steel-CFRP fibre metal laminates. Appl. Compos. Mater..

[49] Múgica J.I., Aretxabaleta L., Ulacia I., Aurrekoetxea J. (Jun. 2014). Impact characterization of thermoformable fibre metal laminates of 2024-T3 aluminium and AZ31B-H24 magnesium based on self-reinforced polypropylene. Compos. Part A Appl. Sci. Manuf..

[50] Jakubczak P., Bieniaś J., Dadej K. (2020). Experimental and numerical investigation into the impact resistance of aluminium carbon laminates. Compos. Struct..

[51] Jakubczak P., Bieniaś J., Droździel M. (Oct. 2020). The collation of impact behaviour of titanium/carbon, aluminum/carbon and conventional carbon fibres laminates. Thin-Walled Struct..

[52] Yao L., Sun G., He W., Meng X., Xie D. (Oct. 2019). Investigation on impact behavior of FMLs under multiple impacts with the same total energy: experimental characterization and numerical simulation. Compos. Struct..

[53] Zhang D., Zhang X., Luo Y., Wang Q. (2018). Experimental study on drop-weight impact response of basalt fiber aluminum laminates (BFMLs). Adv. Mater. Sci. Eng..

[54] Li Z., Zhang J., Jackstadt A., Kärger L. (August 2021, 2022). Low-velocity impact behavior of hybrid CFRP-elastomer-metal laminates in comparison with conventional fiber-metal laminates. Compos. Struct..

[55] Zarei H., Brugo T., Belcari J., Bisadi H., Minak G., Zucchelli A. (2017). Low velocity impact damage assessment of GLARE fiber-metal laminates interleaved by Nylon 6,6 nanofiber mats. Compos. Struct..

[56] Shi Y., Pinna C., Soutis C. (2020). Impact damage characteristics of carbon fibre metal laminates: experiments and simulation. Appl. Compos. Mater..

[57] Ahmadi H., Ekrami M., Sabouri H., Bayat M. (2019). Experimental and numerical investigation on the effect of projectile nose shape in low-velocity impact loading on fiber metal laminate panels. Proc. Inst. Mech. Eng. Part G J. Aerosp. Eng..

[58] Vijayan M., Selladurai V., Kumar V.V. (Mar. 2023). Investigating the influence of nano-silica on low-velocity impact behavior of aluminium-glass fiber sandwich laminate. Silicon.

[59] Lu B., Zhang J., Zheng D., Zhang T., Xie J. (Jan. 2023). Study on off-center impact behavior and damage characterization of carbon fiber reinforced aluminum laminate. Compos. Struct..

[60] Loganathan T.M., Sultan M.T.H., Gobalakrishnan M.K., Muthaiyah G. (2018). Mechanical and Physical Testing of Biocomposites, Fibre-Reinforced Composites and Hybrid Composites.

[61] Kumar V.V., Rajendran S., Balaganesan G., Surendran S., Selvan A., Ramakrishna S. (Aug. 2022). High velocity impact behavior of Hybrid composite under hydrostatic preload. J. Compos. Mater..

[62] Kumar V.V., Rajendran S., Ramakrishna S. (2023). Experimental analysis of ballistic impact on carbon, glass and hybrid composite under hydrostatic prestrain. Adv. Anal. Des. Mar. Struct. - Proc. 9th Int. Conf. Mar. Struct. MARSTRUCT.

[63] Kumar V.V., Rajendran S., Ramakrishna S., Surendran S. (Jun. 2022). Experimental investigation of carbon and glass hybrid composite under ballistic impact for marine applications. Trends Marit. Technol. Eng..

[64] Li K. (Apr. 2023). Soft impact of GLARE fiber metal laminates. Int. J. Impact Eng..

[65] Zhu Z., Li X., Yang R., Xie W., Zhang D. (2023). The energy dissipation mechanism of bi-metal Kevlar\titanium fiber metal laminate under high-velocity impact. Eur. J. Mech. A/Solids.

[66] Zhang R. (Jan. 2023). Ballistic performance of ultralight multifunctional cellular sandwich plates with UHMWPE fiber metal laminate skins. Compos. Struct..

[67] Madika B., Syahrial A.Z. (2024). Study of aluminum/kevlar fi ber composite laminate with and without TiC nanoparticle impregnation and aluminum/carbon fi ber composite laminate for anti-ballistic materials. Int. J. Light. Mater. Manuf..

[68] Mansoori H., Zakeri M., Zarei H.R. (Apr. 2022). A parametric study on the behavior of FMLs based on UHMWPE composite under high velocity impact loading. J. Thermoplast. Compos. Mater..

[69] Gao Y., Shi L., Lu T., Xie W., Cai X. (2024). Ballistic and delamination mechanism of CFRP/aluminum laminates subjected to high velocity impact. Eng. Fract. Mech..

[70] Kosedag E., Aydin M., Ekici R. (Mar. 2022). Effect of stacking sequence and metal volume fraction on the ballistic impact behaviors of ARALL fiber-metal laminates: an experimental study. Polym. Compos..

[71] Shahjouei S., Barati M.R., Tooski M.Y. (2021). High velocity impact response and damage mechanism of an aluminium/glass-carbon fiber/epoxy composite plate reinforced with graphene nano-plates. Fibers Polym..

[72] Rahmani H., Eslami-Farsani R., Ebrahimnezhad-Khaljiri H. (2020). High velocity impact response of aluminum- carbon fibers-epoxy laminated composites toughened by nano silica and zirconia. Fibers Polym..

[73] Muniyan V., Velappan S., Ayyavoo K. (Sep. 2023). Damage behavior and dynamic response of nano-silica reinforced aluminum 2024-T3/GFRP composite laminate subjected to high-velocity impact. J. Reinf. Plast. Compos..

[74] A. D8101 (2017).

[75] Aghamohammadi H., Eslami-Farsani R., Tcharkhtchi A. (2020). The effect of multi-walled carbon nanotubes on the mechanical behavior of basalt fibers metal laminates: an experimental study. Int. J. Adhes. Adhes..

[76] Haro E.E., Odeshi A.G., Szpunar J.A. (2016). The energy absorption behavior of hybrid composite laminates containing nano-fillers under ballistic impact. Int. J. Impact Eng..

[77] Abdullah M.R., Cantwell W.J. (2012). The high-velocity impact response of thermoplastic-matrix fibre-metal laminates. J. Strain Anal. Eng. Des..

[78] Ge F. (Jan. 2023). Ballistic impact response and failure mechanism of aluminum/ultrahigh molecular weight polyethylene fiber laminates with different adhesive quantity. Polym. Compos..

[79] Ahmadi H., Sabouri H., Liaghat G., Bidkhori E. (2011). Experimental and numerical investigation on the high velocity impact response of GLARE with different thickness ratio. Procedia Eng..

[80] Ahmadi H., Liaghat G.H., Sabouri H., Bidkhouri E. (2013). Investigation on the high velocity impact properties of glass-reinforced fiber metal laminates. J. Compos. Mater..

[81] Yaghoubi A.S., Liaw B. (Oct. 2013). An experimental and numerical investigation of thickness effect on cross-ply GLARE 5 FML plates subjected to ballistic impact. ASME Int. Mech. Eng. Congr. Expo. Proc..

[82] Kaboglu C. (2018). High-velocity impact deformation and perforation of fibre metal laminates. J. Mater. Sci..

[83] Li X., Yahya M.Y., Bassiri Nia A., Wang Z., Lu G. (2016). Dynamic failure of fibre-metal laminates under impact loading - experimental observations. J. Reinf. Plast. Compos..

[84] Vlot A. (1996). Impact loading on fibre metal laminates. Int. J. Impact Eng..

[85] Seyed Yaghoubi A., Liaw B. (2013). Effect of lay-up orientation on ballistic impact behaviors of GLARE 5 FML beams. Int. J. Impact Eng..

[86] Khan S.H., Sharma A.P. (2022). Influence of metal/composite interface on the damage behavior and energy absorption mechanisms of FMLs against projectile impact. Def. Technol..

[87] Ramadhan A.A., Abu Talib A.R., Mohd Rafie A.S., Zahari R. (2013). High velocity impact response of Kevlar-29/epoxy and 6061-T6 aluminum laminated panels. Mater. Des..

[88] Sharma A.P., Velmurugan R., Shankar K., Ha S.K. (2021). High-velocity impact response of titanium-based fiber metal laminates. Part II: analytical modeling. Int. J. Impact Eng..

[89] Sharma A.P., Velmurugan R., Shankar K., Ha S.K. (Jun. 2021). High-velocity impact response of titanium-based fiber metal laminates. Part I: experimental investigations. Int. J. Impact Eng..

[90] Sharma A.P., Velmurugan R. (Jan. 2023). High-velocity impact response of titanium/composite laminates: an analytical modeling. Compos. Mater. High Strain Rate Stud..

[91] Reyes V.G., Cantwell W.J. (2004). The high velocity impact response of composite and FML-reinforced sandwich structures. Compos. Sci. Technol..

[92] Ansari M.M., Chakrabarti A. (2017). Influence of projectile nose shape and incidence angle on the ballistic perforation of laminated glass fiber composite plate. Compos. Sci. Technol..

[93] Mansoori H., Zakeri M., Guagliano M. (2022). Energy absorption and damage mechanism of UHMWPE-aluminum composite sandwich laminate under impact loading: an experimental investigation. J. Sandw. Struct. Mater..

[94] Majzoobi G., Kashfi M., Keshavarzan M., Riazalhosseini M. (Feb. 2022). Effect of projectile nose on high-velocity impact behavior of fiber metal laminates. Polym. Compos..

[95] Gaur B., Patel M., Patel S. (Feb. 2023). Strain rate effect on CRALL under high-velocity impact by different projectiles. J. Brazilian Soc. Mech. Sci. Eng..

[96] Suresh Kumar S., Shankar P.A., Lalith Kumar K. (Jun. 2022). Failure investigation on high velocity impact deformation of boron carbide (B4C) reinforced fiber metal laminates of titanium/glass fiber reinforced polymer. Def. Technol..

[97] Chen Y., Pang B., Zheng W., Peng K. (Dec. 2013). Experimental investigation on normal and oblique ballistic impact behavior of fiber metal laminates. J. Reinf. Plast. Compos..

[98] Sangsefidi M., Sabouri H., Mir M., Hasanpour A. (Feb. 2021). High-velocity impact response of fiber metal laminates: experimental investigation of projectile's deformability. Thin-Walled Struct..

[99] Abdullah M.R., Cantwell W.J. (Sep. 2006). The impact resistance of polypropylene-based fibre–metal laminates. Compos. Sci. Technol..

[100] Majzoobi G.H., Morshedi H., Farhadi K. (2018). The effect of aluminum and titanium sequence on ballistic limit of bi-metal 2/1 FMLs. Thin-Walled Struct..

[101] Li X., Zhang X., Guo Y., Shim V.P.P.W., Yang J., Chai G.B. (Apr. 2018). Influence of fiber type on the impact response of titanium-based fiber-metal laminates. Int. J. Impact Eng..

[102] Pang Y., Yan X., Wu L., Qu J. (Jan. 2022). Experiment study of basalt fiber/steel hybrid laminates under high-velocity impact performance by projectiles. Compos. Struct..

[103] Sharma A.P., Khan S.H. (2018). Influence of metal layer distribution on the projectiles impact response of glass fiber reinforced aluminum laminates. Polym. Test..

[104] Bahari-Sambran F., Eslami-Farsani R., Arbab Chirani S. (2020). The flexural and impact behavior of the laminated aluminum-epoxy/basalt fibers composites containing nanoclay: an experimental investigation. J. Sandw. Struct. Mater..

[105] Liu C., Zhang Y.X., Ye L. (2017). High velocity impact responses of sandwich panels with metal fibre laminate skins and aluminium foam core. Int. J. Impact Eng..

[106] Yelamanchi B., Macdonald E., Gonzalez-Canche N.G., Carrillo J.G., Cortes P. (2020). The mechanical properties of fiber metal laminates based on 3d printed composites. Materials.

[107] Yelamanchi B., MacDonald E., Gonzalez-Canche N.G., Carrillo J.G., Cortes P. (Nov. 2023). The fracture properties of fiber metal laminates based on a 3D printed glass fiber composite. J. Thermoplast. Compos. Mater..

[108] Ishak N.M., Malingam S.D., Mansor M.R., Razali N., Mustafa Z., Ghani A.F.A. (2021). Investigation of natural fibre metal laminate as car front hood. Mater. Res. Express.

